# Coordinated modulation of long non-coding RNA ASBEL and curcumin co-delivery through multicomponent nanocomplexes for synchronous triple-negative breast cancer theranostics

**DOI:** 10.1186/s12951-023-02168-8

**Published:** 2023-10-31

**Authors:** Xuesong He, Fengjuan Lin, Runqing Jia, Yang Xia, Zhaoyuan Liang, Xiangqian Xiao, Qin Hu, Xiongwei Deng, Qun Li, Wang Sheng

**Affiliations:** 1 Department of Environment and Life Science, Beijing International Science and Technology, Cooperation Base of Antivirus Drug, Beijing University of Technology, Beijing, 100124 China; 2 Department of Oncology, School of Medicine, Shanghai East Hospital, Tongji University, Shanghai, 200123 China; 3 College of Life Science and Bioengineering, Beijing University of Technology, Beijing, 100049 China

**Keywords:** LncRNA ASBEL, Curcumin, Polyelectrolyte nanocomplexes, Self-assembled, Triple-negative breast cancer, Synchronous theranostics

## Abstract

**Background:**

Abnormally regulated long non-coding RNAs (lncRNAs) functions in cancer emphasize their potential to serve as potential targets for cancer therapeutic intervention. LncRNA ASBEL has been identified as oncogene and an anti-sense transcript of tumor-suppressor gene of BTG3 in triple-negative breast cancer (TNBC).

**Results:**

Herein, multicomponent self-assembled polyelectrolyte nanocomplexes (CANPs) based on the polyelectrolytes of bioactive hyaluronic acid (HA) and chitosan hydrochloride (CS) were designed and prepared for the collaborative modulation of oncogenic lncRNA ASBEL with antago3, an oligonucleotide antagonist targeting lncRNA ASBEL and hydrophobic curcumin (Cur) co-delivery for synergetic TNBC therapy. Antago3 and Cur co-incorporated CANPs were achieved via a one-step assembling strategy with the cooperation of noncovalent electrostatic interactions, hydrogen-bonding, and hydrophobic interactions. Moreover, the multicomponent assembled CANPs were ulteriorly decorated with a near-infrared fluorescence (NIRF) Cy-5.5 dye (FCANPs) for synchronous NIRF imaging and therapy monitoring performance. Resultantly, MDA-MB-231 cells proliferation, migration, and invasion were efficiently inhibited, and the highest apoptosis ratio was induced by FCANPs with coordination patterns. At the molecular level, effective regulation of lncRNA ASBEL/BTG3 and synchronous regulation of Bcl-2 and c-Met pathways could be observed.

**Conclusion:**

As expected, systemic administration of FCANPs resulted in targeted and preferential accumulation of near-infrared fluorescence signal and Cur in the tumor tissue. More attractively, systemic FCANPs-mediated collaborative modulating lncRNA ASBEL/BTG3 and Cur co-delivery significantly suppressed the MDA-MB-231 xenograft tumor growth, inhibited metastasis and extended survival rate with negligible systemic toxicity. Our present study represented an effective approach to developing a promising theranostic platform for combating TNBC in a combined therapy pattern.

**Supplementary Information:**

The online version contains supplementary material available at 10.1186/s12951-023-02168-8.

## Introduction

Unresectable locally advanced or metastatic triple-negative breast cancer (TNBC) is the most notoriously aggressive breast cancer type with poor outcomes over the past decade, which lacks expression of progesterone, Her2/Neu and estrogen receptors [1, 2]. Conventional cytotoxic chemotherapy remains the mainstay of treatment intervention for TNBC patients in the neoadjuvant, adjuvant or metastatic settings. However, the therapeutic effect is not prominent due to the frequently developed chemoresistance, visceral metastases and universal recurrence [3, 4]. High rates of visceral metastases in TNBC patients caused a relatively shorter median survival time. More seriously, TNBC patients are unsusceptible to targeted or hormonal therapies due to the “triple-negative” characteristic [5]. As our understanding of TNBC continues to advance, it has become increasingly clear that treatments need to address the complexity and heterogeneity of the disease. Thus, innovative therapy strategy based on novel molecular targets associated with the biological characteristics of TNBC is currently vigorously pursued [6].

Whole genome and transcriptome sequencing technologies have revealed that 98% of transcriptional products are non-coding RNAs [7, 8]. In past decades, long non-coding RNA (lncRNA) defined as a class of non-protein-coding RNA molecules longer than 200 nucleotides, has been shown to participate in almost all cellular processes [9–11]. Moreover, lncRNAs play important roles in many human diseases including cancer, which are widely reported to regulate tumor cell growth, migration, invasion, and apoptosis [12–15]. Importantly, lncRNAs display a tissue-specific expression pattern, and aberrant expression of various lncRNA has been observed in nearly every type of cancer, directly or indirectly regulating tumorigenesis, acting as oncogenic or antioncogenic lncRNA [12–14, 16]. Thus, abnormally regulated lncRNAs in diverse cancers have served as potential targets for therapeutic intervention [17–20]. The transcription of the lncRNA ASBEL has been identified as a natural antisense transcript (NATs) of B-cell translocation gene 3 (BTG3) locus. ASBEL involves in tumorigenesis of various cancers, including TNBC [21–23]. It has been identified that ASBEL forms duplexes with BTG3 mRNA and negatively regulates the levels of BTG3 gene. BTG3 belongs to the BTG/transducer of the ErbB2 family that exhibits antiproliferative function. BTG3 has been well identified as a tumor suppressor gene that could inhibit proliferation, migration and invasion in a variety of cancer types [24]. Moreover, studies have confirmed that the upregulation of the BTG3 gene in cancer cells could enhance the toxicity of chemotherapy drugs, including cisplatin and paclitaxel [25]. Therefore, lncRNA ASBEL has been recognized as an oncogenic lncRNA and a potential target for cancer therapies. Nevertheless, modulating lncRNA expression with conventional methods remains a challenge. Double-stranded (ds) small interfering RNA (siRNA) could be used to knockdown specific lncRNA through degradation of target sequence. However, many studies showed that the efficiency of targeting lncRNA with siRNA is limited due to the off-target effects [26]. In addition, the secondary structure of the lncRNA might preclude or limit optimal association with a siRNA. To address this need, our laboratory previously screened a series of single-stranded oligonucleotide antagonists and identified a specific antago3 for lncRNA ASBEL knockdown more efficacy than siRNA strategy. Furthermore, antago3 exhibited certain anticancer activity for combating TNBC both in vitro and in vivo [27]. Nevertheless, the naked single-stranded oligonucleotide of antago3 is prone to be degraded by endogenous enzymes in vivo and intracellular barriers limit its direct use. Therefore, an efficient delivery system should be developed for lncRNA ASBEL knockdown and TNBC treatment.

Curcumin (Cur), a natural and hydrophobic polyphenolic compound derived from turmeric (Curcuma) longa, has exhibited diverse pharmacologic effects, including anti-oxidant, anti-inflammatory, anti-Alzheimer, anti-depression and anti-cancer properties [28–30]. Cur can induce apoptosis, and prevent cell proliferation, metastasis and angiogenesis via targeting several defined signal transduction pathways [31, 32]. In addition, recent findings have confirmed that Cur could regulate miRNAs and lncRNAs expression in a variety of cancers [33–35]. However, the low aqueous solubility, poor bioavailability, poor absorption and systemic elimination hinder Cur usage as a potential anti-cancer agent [37]. In addition, despite Cur showed certain chemo-preventive effects and therapeutic value in the treatment of TNBC, the efficiency of the monotherapy pattern by Cur is limited due to the heterogeneity and complexity of TNBC and the inherent resistance of small molecule drugs [36–39]. A combination of two or more therapeutic agents targeting different pathways or mechanisms has been explored to raise the chances of eliminating cancer [40–44]. How to effectively deliver biomacromolecule and small molecule drugs with different physicochemical properties to realize combinational behavior has always been a challenge and a subject of active research.

Self-assembled polyelectrolyte nanocomplexes (SPECs) typically formed by two or more oppositely charged polyelectrolytes [45], represent a facile and straightforward way to generate advanced nanostructures from surface nano-coating to self-assembled nanoparticles depending on the building blocks and parameters [46–48]. Integrating proper crosslinkers and cargos, multicomponent SPECs can be established by the cooperation of various secondary attractive interactions among the polyelectrolytes, bridging linkers and cargos, including electrostatic interactions, hydrogen-bonding and hydrophobic interaction [49–52]. Ideally, homogeneous nanocomplexes with optimal size, surface properties, colloidal stability and cargo loading efficiency could be constructed by regulating the preparation parameters [53]. In addition, the preparation process of SPECs is economical and convenient, and can realize the full utilization of building polyelectrolytes [54]. SPECs strategy has been utilized to incorporate both hydrophilic and hydrophobic drugs. Hyaluronic acid (HA) is a negatively charged biologically-active natural glycosaminoglycan present in the extracellular matrix and synovial fluids. HA has been extensively studied in nanomedicine and biomedical application due to its good biological activities. Moreover, HA backbone could specifically bind to HA receptors overexpressed on the surface of various cancer cells such as CD44, RAHMN and LYVE-1 [55]. 80–90% of TNBC cells express CD44, using HA as a CD44 targeted drug delivery agent is a good choice for TNBC therapy [56]. On the other hand, the cationic polysaccharide of chitosan (CS) partially deacetylated form of chitin, has been broadly used as another candidate bioactive material in developing nanomedicine. Specially, the positive charge of CS endows it superior function for gene complex, delivery and therapy in a variety of diseases. Noteworthy, as biologically-active natural materials, both CS and HA have excellent biocompatibility and biodegradability. Hence, HA-CS based bioactive SPECs are poised to be developed for various applications in nanomedicine and tissue engineering [57].

**Scheme 1:**
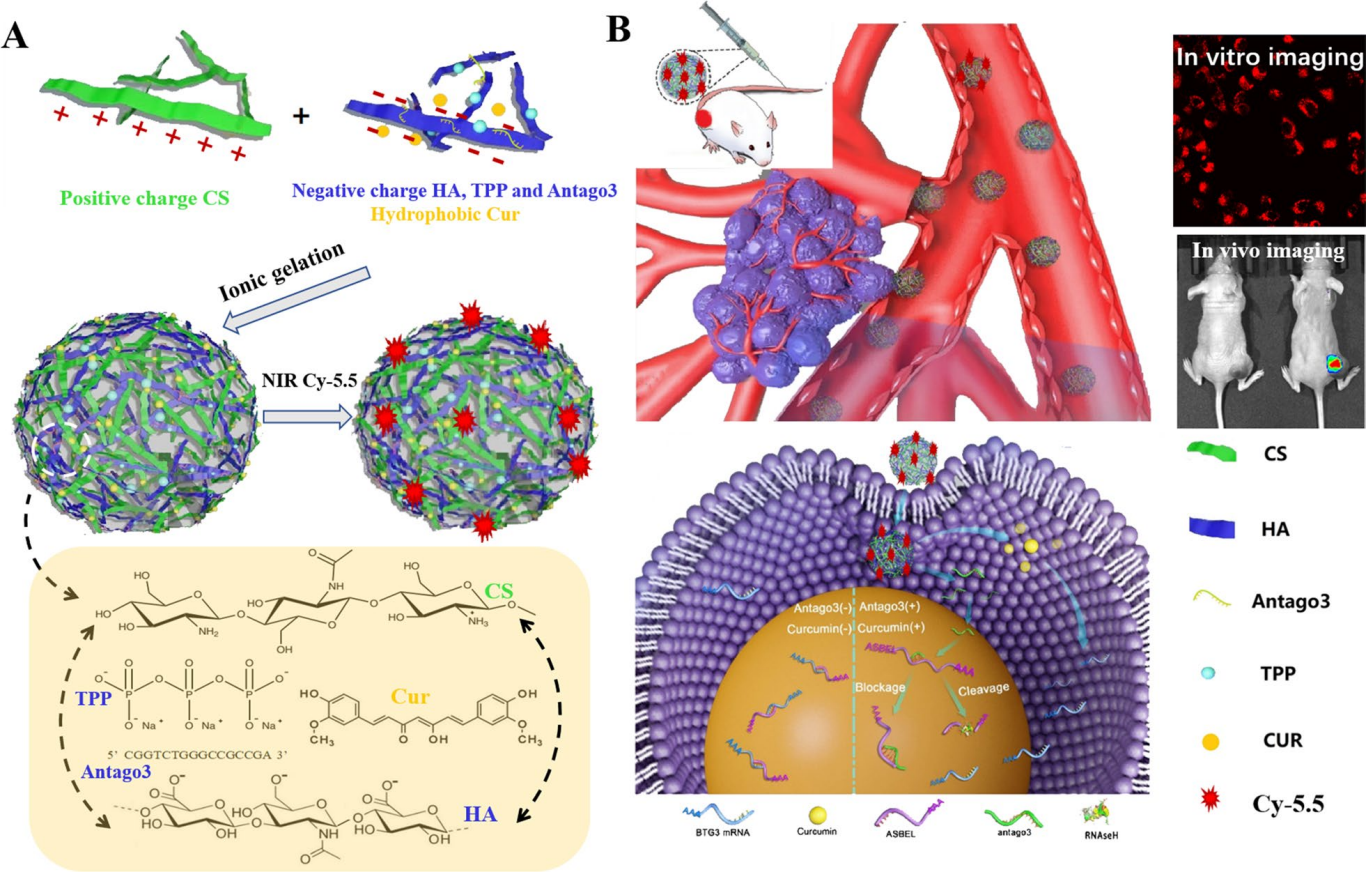
Design and synergistic anticancer effects of FCANPs containing antago3 and Cur, and surface modified with NIR fluorescence. **A** Schematic outlining the assembly process of HA-CS-based nanocomplexes and the strategy for the co-incorporation of antago3 and Cur. The chemical structure formula of HA, CS, TPP, Cur and the sequence of antago3 were presented. **B** FCANPs enable targeted collaborative modulation of lncRNA ASBEL/BTG3 with antago3 and Cur co-delivery for synchronous TNBC theranostics

ASBEL is a target of Wnt/β-catenin signaling which is correlated with tumorigenesis, metastasis, cancer stemness, and poor prognosis in TNBC patients, and can be inhibited by antago3. Previous studies have shown that Curcumin inhibits the canonical Wnt/β-catenin pathway. Thus, both antago3 and Curcumin have synergetic effect in cancer therapy through Wnt/β-catenin signaling [58, 59]. Taking all the above into consideration, herein we reported the development of HA and CS as parent bioactive materials to realize feasible multicomponent self-assembly in fabricating antago3 and Cur co-incorporated CANPs for the synergetic treatment of TNBC. Meanwhile, the resulting CANPs were surface decorated with a near-infrared fluorescence (NIRF) Cy-5.5 dye (FCANPs) for further synchronous imaging and therapy monitoring. (Scheme 1A). Antago3 and Cur could be efficiently incorporated into FCANPs based on the cooperation of electrostatic noncovalent interactions, hydrogen-bonding and hydrophobic interactions. In vitro studies showed that antago3 and Cur could be efficiently delivered into MDA-MB-231 cancer cells via FCANPs and achieved synergetic anti-cancer effects, which inhibited cell proliferation, migration/invasion as well as induced apoptosis effectively. At the molecular level, effective down-regulation of lncRNA ASBEL with antago3 resulted in the upregulation of BTG3 gene and synchronous regulation of Bcl-2 and c-Met associated anti-cancer signal pathways. Systemic administration of the FCANPs resulted in targeted and preferential accumulation of NIR fluorescence and antago3/Cur in the tumor tissue, elicited a higher therapeutic efficacy than Cur and antago3 monotherapy, inhibited tumor metastasis and prolonged survival of the MDA-MB-231 tumor-bearing animals with good biocompatibility (Scheme 1B). As far as we know, the combined treatment with small molecule drug and lncRNA modulation via nanoplatform delivery system for synergistic anti-TNBC therapy has not been reported. This study can provide a way to combine the effective ingredients of traditional Chinese medicine with anti-sense DNA oligonucleotides through nano-based drug delivery system in cancer therapy.

## Results and discussion

### Preparation and physicochemical characterization of nanocomplexes

The identification of abnormally regulated lncRNA provides opportunities to develop novel therapeutics for cancers that are based on targeting lncRNA. Previously, we demonstrated upregulated lncRNA ASBEL in MDA-MB-231 cells and it was associated with cell proliferation, invasion/migration and apoptosis. Furthermore, our laboratory screened a series of single-stranded oligonucleotide antagonists and identified a specific antago3 (5′-CGGTCTGGGCCGCCGA-3′) to knockdown lncRNA ASBEL and treat TNBC both in vitro and in vivo [27]. Despite Cur showed chemo-preventive effects and therapeutic value in the treatment of TNBC, single Cur therapy effect is limited due to the heterogeneity and complexity of TNBC. In the present study, we demonstrated self-assembled nanocomplexes based on the bioactive polyelectrolytes of HA and CS for the collaborative modulation of oncogenic lncRNA ASBEL with antago3 and Cur co-delivery (CANPs) for synergetic TNBC therapy. Scheme 1A depicts the assembling process of Cur and antago3 co-incorporated CANPs and then surface decorated with NIR Cy-5.5 dye (FCANPs). Electrostatic interactions between oppositely charged polyelectrolytes of HA and CS drive the formation of HA-CS NPs. TPP, a small ion with triple negative charge, was used as the physical crosslinking agent to stabilize the NPs. In addition, hydrogen bonds, hydrophobic interaction and other intermolecular forces also promote to stable the NPs to a certain extent. Recent studies have reported on the incorporation of hydrophobic drugs into SPECs and confirmed the formation of hydrophobic cavities in the structure of SPECs [60]. The properties of formed HA-CS NPs greatly depend on the ratios of the two components and methods of mixing. The mass ratio of CS to HA of 1:2 was selected in this study as our previous work has proven that this ratio was suitable to obtain the optimal NPs with proper size, zeta potential, production yield and satisfied stability [55]. Different mass ratios of the four components (CS, HA, Cur and antago3) were assembled to optimize construction conditions by measuring the EE and LC. As shown in Table 1, relative high EE% of antago3 (> 80%) was found in all experimental parameters, demonstrating the high incorporation efficiency of antago3 in the nanocomplexes, which could be ascribed to the strong electronic interactions between positively charged CS and the antago3. In contrast, the EE% and LC% of Cur were comparatively low (40–70%) in all the tested ratios. On account of the highly hydrophobic property of Cur, it can only be embedded into the hydrophobic area, thus resulting in a relatively low entrapment efficiency. Blank NPs (BNPs) and other NPs carrying only Cur (CNPs) and antago3 (ANPs) were also prepared.

**Table 1:** Effects of mass ratios of various components on embedding efficiencies % (EE) and loading contents % (LC) of Cur and antago3

HA:CS:antago3:Cur	2:1:0.05:0.05	2:1:0.075:0.075	2:1:0.1:0.1	2:1:0.15:0.15	2:1:0.2:0.2
EE % of antago3	97.1	93.2	90.1	87.2	81.1
LC % of antago3	2.26	3.22	4.11	5.86	7.16
EE% of Cur	70.3	66.5	58.2	51.2	43.1
LC% of Cur	1.64	2.32	2.70	3.52	3.93

**Fig. 1:**
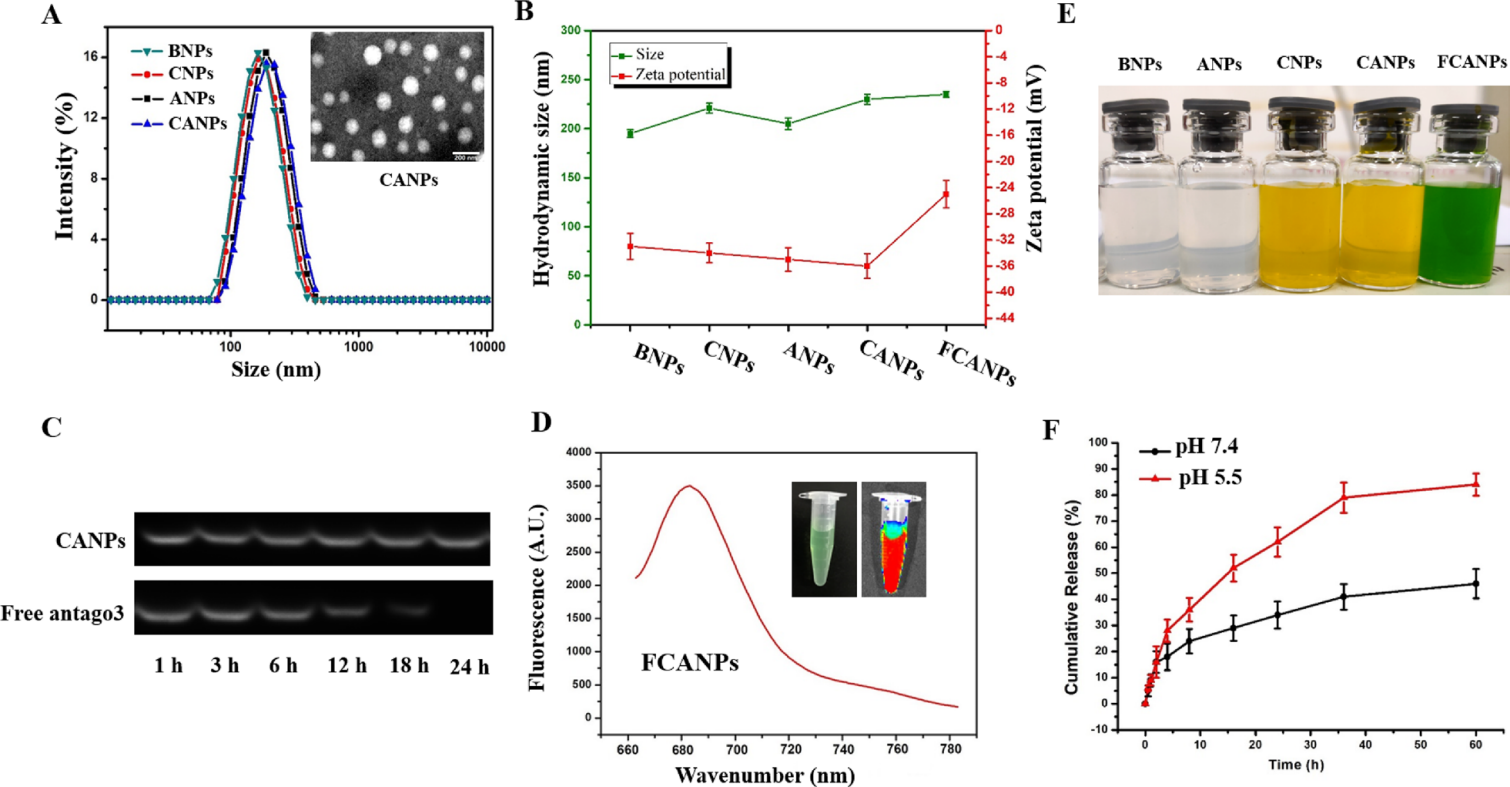
**A** Size distribution histogram BNPs, CNPs, ANPs and CANPs and TEM images (inset) of CANPs. Scale bar: 200 nm. **B** Change in the particle size and zeta potential of BNPs. CNPs, ANPs, CANPs and FCANPs. **C** The antago3 stability test under the presence of DNase I. **D** Fluorescent spectra of FCANPs. The inset in **D** shows the bright field and fluorescent images of FCANPs. **E** Representative photographs of BNPs. CNPs, ANPs, CANPs and FCANPs dispersed in aqueous solutions. **F** Cumulative Cur release from FCANPs at 37 °C in PBS of pH 7.4 and 5.5

Various kinds of NPs with average size predominantly between 190 and 230 nm were reflected by DLS measurements (Fig. 1A, B). On account of the similar average size of ANPs to that of BNPs, we speculated that incorporation of antago3 into ANPs had a negligible effect on the size. In contrast, CNPs and CANPs showed an increase in size (≈ 220 nm) compared with BNPs (≈ 190 nm) by DLS measurements, suggesting that such an increase was correlative to the incorporation of Cur in the formulations. As revealed by TEM analysis, BNPs, ANPs, CNPs and CANPs all exhibited spherical morphology with uniform size distributions and without aggregation (Fig. 1A and Additional file 1: Fig. S1). The results of TEM measurements exhibited smaller diameter than that detected by DLS measurements, which could be ascribed to the distinction between the dehydration of NPs corona in dry state by TEM analysis and the hydrodynamic swelling state of the samples suspended in aqueous media by DLS measurements. All formulations showed a similarly negative z-potential (in the region of − 33 mV) that confirmed the surface presence of ionized carboxylic groups of HA. Z-potential of ANPs (− 34 mV) and CANPs (− 36 mV) was lower than that of BNPs (− 32 mV) and CNPs (− 33 mV) (Fig. 1B). The change in surface zeta potential could be originated from the negative charged characterization of antago3. Furthermore, we investigated the stability of CANPs based on the the particle size and loaded Cur in the CANPs after 2 weeks of storage at room temperature. As shown in Additional file 1: Table S1, the size of CANPs increased slightly during the storage period and up to 2 weeks. Although the EE% of Cur decreased slightly along with storage time, the overall EE% of Cur could also maintain comparative quantity in comparison with the initial state. We ascribed this phenomenon to the diffusion of small amount of Cur from CANPs during the storage time. Overall, the results manifested that CANPs could maintain stability considering the size and the state of loaded Cur. As oligonucleotide antagonists is vulnerable to DNase in the biological environments, we next investigated if CANPs could protect loaded antago3 from degradation by DNase. Correspondingly, naked antago3 and CANPs containing equal amount of antago3 were incubated in DNase I-containing PBS for different time points. As displayed in Fig. 1C, the band intensity of naked antago3 gradually reduced and almost disappeared after 24 h incubation. In contrast, no obvious antago3 degradation under the protection of CANPs could be observed after 24 h of incubation, suggesting that the incorporation of antago3 in CANPs could sterically hinder the access of nucleases to the antago3. Such protective ability provided by CANPs was significant important for in vivo application.

In an effort to develop a theranostic system, we therefore prepared NIRF dye Cy-5.5 conjugated BNPs (FBNPs) and CANPs (FCANPs) through mild reaction between amine group of Cy-5.5-NH_2_ and carboxyl group of HA. An increase in the zeta potential of FCANPs (− 24 mV) compared with CANPs was observed, while it had negligible influence on the size of FCANPs compared with CANPs (Fig. 1B). Upon successful conjugation of Cy-5.5 molecules, the colloidal solution of FCANPs turned green and its max emission at 678 nm with a strong NIRF signal was observed (Fig. 1D, E). Infrared spectroscopic and zeta potential characterization both confirmed the conjugation of Cy-5.5 molecules to the surface of FCANPs. Adsorption of plasma proteins will greatly influence the in vivo performance of nanoparticles. Therefore, we next used BSA as a model protein to test the protein adsorption ability of constructed FCANPs. As displayed in Additional file 1: Fig S2, negative charged FCANPs showed minimal protein adsorption after different times co-incubation (0.5, 1, 2, 4 and 6 h), indicating FCANPs could avoid nonspecific interaction with serum protein. Due to the hydrophobic nature of Cur, the in vitro release profile of Cur from FCANPs was investigated in sink conditions of PBS containing 1% Tween-80. pH 5.5 was used mimicking the intracellular lysosomal compartment acidic environment. As can be seen in Fig. 1F, about 26% and 50% of Cur can be released within the first 24 h at pH 7.4 and 5.5, respectively. As time goes on to 60 h, the cumulative Cur release reached to nearly 80% at pH 5.5 and 40% at pH 7.4. The calculated results revealed that the release of Cur at both pH 5.5 was distinctly faster than that at pH 7.4, suggesting that acidic condition increased the release rate of Cur compared with neutral condition. The delayed Cur release in pH 7.4 medium and the acid-accelerated release in pH 5.5 would be beneficial to tumor therapy.

### In vitro cellular uptake assays

**Fig. 2:**
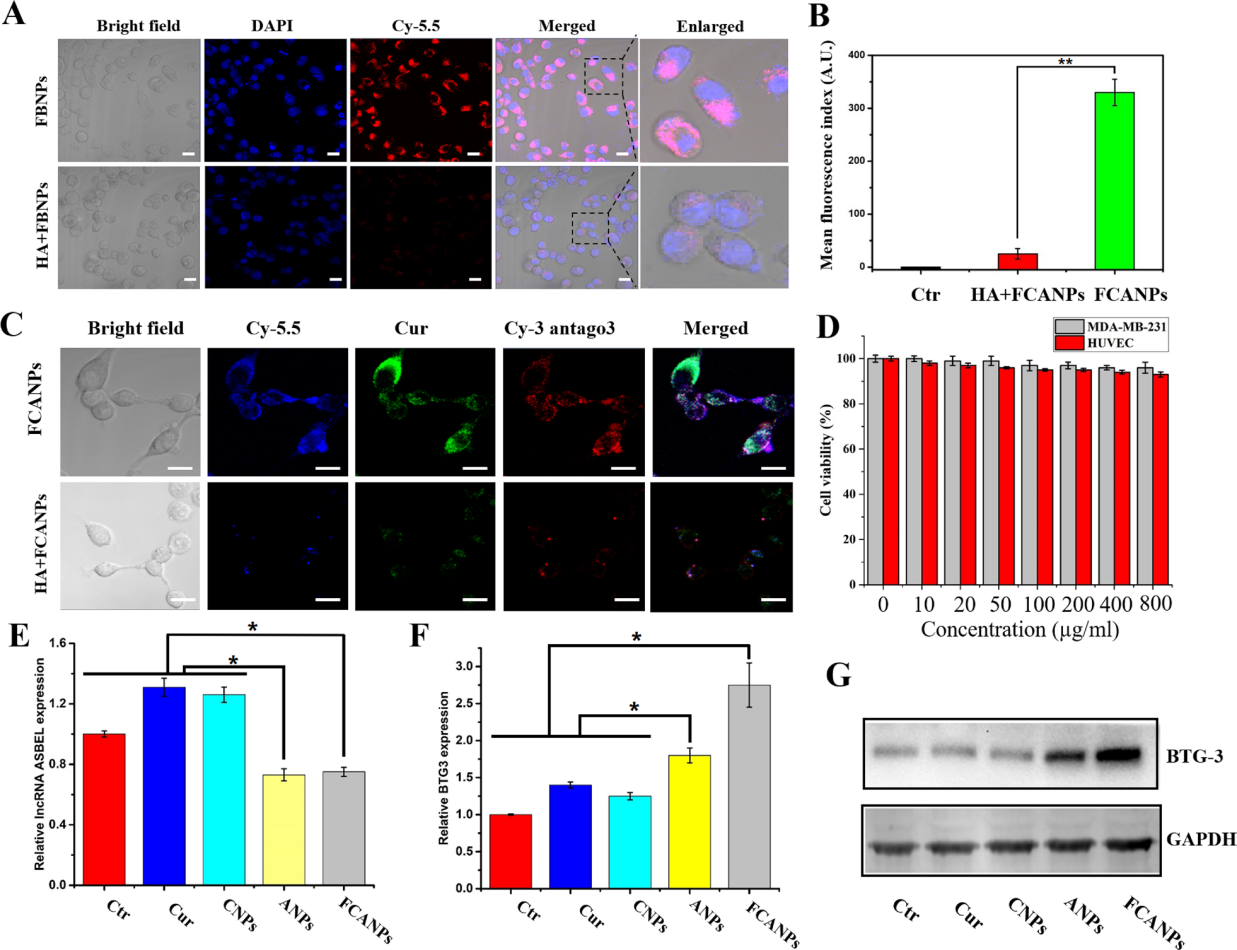
**A** Subcellular internalization and localization of FBNPs in MDA-MB-231 cancer cells with or without the pretreatment of excess HA. **B** The relative fluorescence indexes. **C** Subcellular internalization and localization of FCANPs in MDA-MB-231 cancer cells with or without the pretreatment of excess HA. **D** Viability of MDA-MB-231 and HUVEC cells after treatment with various concentrations of BNPs (*n* = 3). There were no significant differences between the two groups. **E**, **F** Relative expression of lncRNA ASBEL and BTG3 after various treatments by qRT-PCR analysis. **G** BTG3 protein expression after various treatments by western-blot analysis with GAPDH as an internal protein. Scale bar: 25 μm. Data are shown as mean ± SD, n = 3, Statistical significance was calculated by one-way analysis of variance (ANOVA). *p < 0.05; **P < 0.01

As we know, HA-based nanomedicine can specifically bind to various cancer cells including MDA-MB-231 cells due to the over-expressed receptors on the surface such as CD44, RAHMN and LYVE-1 [61]. Thus, intracellular fluorescence imaging of FBNPs in MDA-MB-231 cells was firstly investigated by CLSM. As shown in Fig. 2A, the cells displayed high Cy-5.5 fluorescence signal in the cytoplasm after 4 h incubation with FBNPs. To further explore the potential mechanism, the cells were pretreated with redundant HA to block HA receptors before cellular uptake experiment. In sharp contrast, the intracellular fluorescence intensity of Cy-5.5 of FCANP plus HA group was significantly reduced compared with FCANP only group (Fig. 2B). It can be explained that free HA bind to HA-receptors in advance resulting in the decreased cellular uptake of FCANPs. Moreover, we labeled antago3 with Cy-3 fluorescent dye to visualize antago3 delivery in order to distinguish between the fluorescence of Cur and Cy-5.5. The results suggested that FCANPs could efficiently deliver Cur and antago3 into the cytoplasm of MDA-MB-231 cells. In addition, HA block assay also reflected HA receptor-mediated endocytosis is a major mechanism responsible for the uptake of FCANPs by MDA-MB-231 cells. (Fig. 2C). As an excellent NPs, the NPs themselves must exhibit low cytotoxicity. As shown in Fig. 2D, the BNPs exhibited negligible toxicity in both MDA-MB-231 and HUVEC cells. The low cytotoxicity of FBNPs might be due to the high biocompatibility of HA and CS. Antago3, as a specific oligonucleotide antagonist for lncRNA ASBEL knockdown, we next evaluated the transcriptional level of lncRNA ASBEL by qRT-PCR after different formulations treatment in MDA-MB-231 cells. As presented in Fig. 2E, decreased level of lncRNA ASBEL was observed in ANPs- and FCANPs-treated cells compared with other three groups. We also found that Cur and CNPs treatment could result in elevated expression of lncRNA ASBEL in MDA-MB-231 cells. This result was consistent with recent studies confirming that Cur could regulate lncRNA expression in cancer cells. As a possible mechanism underlying lncRNA ASBEL has been identified as natural antisense transcript of BTG3, we next examined the expression of BTG3 gene and protein. Upregulation of BTG3 was found both in ANPs- and FCANPs-treated cells compared to other three groups at both mRNA (Fig. 2F) and protein levels (Fig. 2G). BTG3 has been identified as a tumor suppressor gene that could inhibit proliferation, migration/invasion, metastasis and angiogenesis in a variety of cancers [62].

### In vitro biological function and anti-cancer activities

**Fig. 3:**
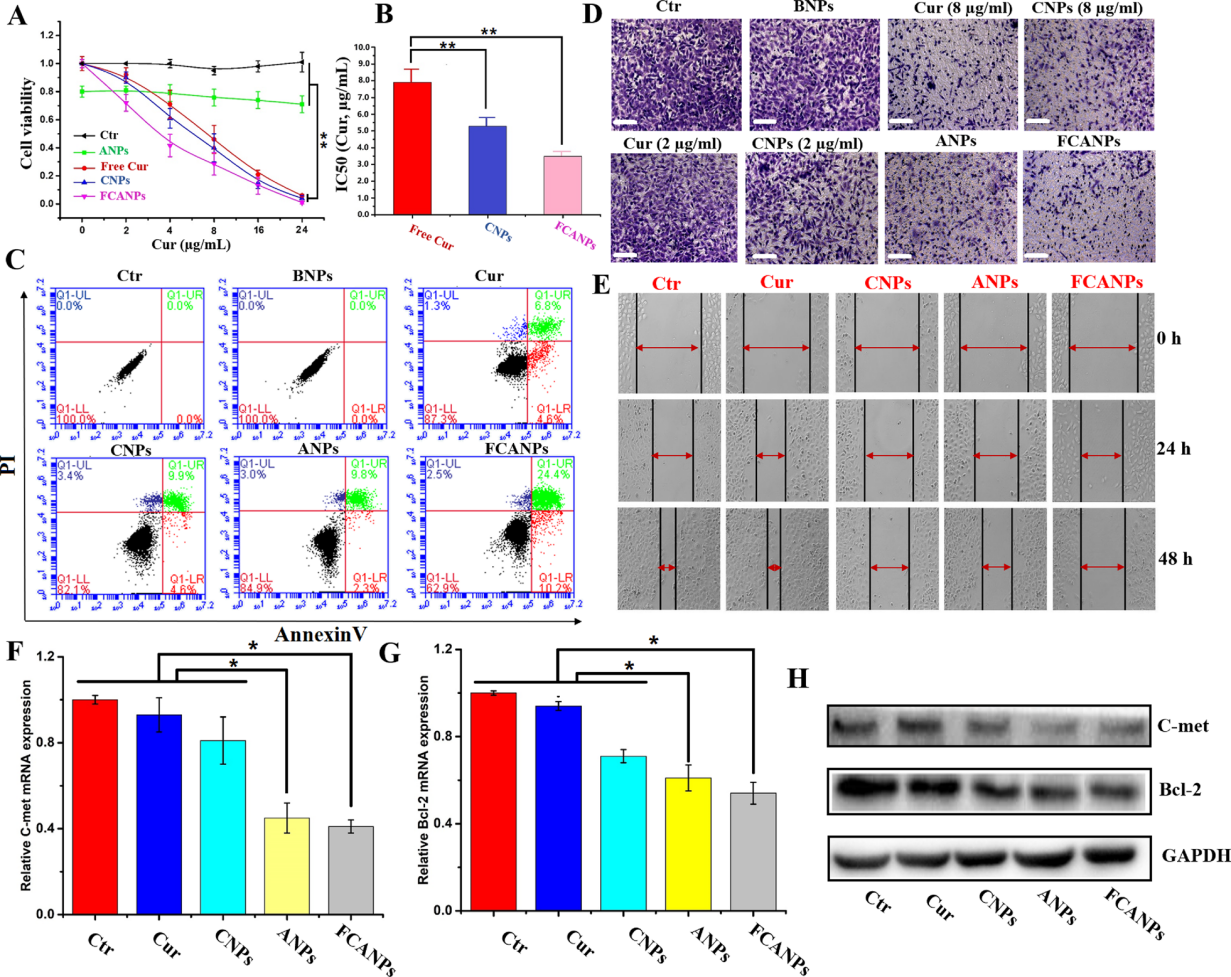
**A** In vitro cytotoxicity of MDA-MB-231 cells treated with saline, free Cur, ANPs, CNPs and CANPs at different concentrations of Cur for 48 h. **B** Relative IC 50 of free Cur, CNPs and CANPs to MDA-MB-231 cells based on Cur. Scale bar 100 µm. **C** Apoptosis-inducing effects with different formulations on MDA-MB-231 cells by flow cytometric analysis. **D** Matrigel trans-well assays in MDA-MB-231 cells after various treatments. Scale bar 100 µm. **E** Wound healing assays in MDA-MB-231 cells after various treatments. **F**, **G** Relative c-Met and Bcl-2 expression after different treatments by qRT-PCR analysis. **H** Relative C-met and Bcl-2 expression after different treatments by western-blot analysis. Data are shown as mean ± SD, n = 3. Statistical significance was calculated by one-way analysis of variance (ANOVA). *p < 0.05, **p < 0.01. In the apoptosis, transwell, wound healing, qRT-PCR and western-blot assays, free Cur, CNPs and FCANPs were used at 2 μg/mL based on the dose of Cur. ANPs and FCANPs were used at 100 nM based on the dose of antago3

We and other groups have demonstrated that knockdown of lncRNA ASBEL could inhibit cell viability, migration/invasion and induce apoptosis in various types of cancer cells through upregulating BTG3 expression. Upregulation of BTG3 was closed linked to the tumorigenesis of various cancers [63]. Thus, the main goal of our current study is that whether targeted and combination modulation of lncRNA ASBEL/BTG3 with antago3 and Cur co-delivery by FCANPs would result in enhanced therapy efficiency in MDA-MB-231 cells due to their synergistic effects. Therefore, the biological effects of FCANPs-mediated co-delivery of antago3 and Cur in MDA-MB-231 cells were systematically investigated. First of all, we undertook CCK-8 assays to evaluate if the co-delivery of antago3 and Cur will result in enhanced cell cytotoxicity. MDA-MB-231 cells were incubated with saline, free Cur, CNPs, ANPs and FCANPs at different Cur concentrations ranging from 0 to 24 µg/mL. As presented in Fig. 3A, ANPs treatment (100 nM of antago3) showed reduced cell viability compared to the control group, indicating that ANPs-mediated antago3 delivery could inhibit the proliferation of MDA-MB-231 cells. Free Cur and CNPs exhibited obvious cytotoxicity to MDA-MB-231 cells and showed a concentration-dependent behavior. In addition, Cur-loaded CNPs displayed slightly higher cytotoxicity than free Cur, which could be ascribed to the CNPs-mediated targeted Cur delivery. Furthermore, the cells treated with FCANPs resulted in lower cell survival rate compared to those treated with free Cur or CNPs at an equivalent dose of Cur, indicating that the combination of antago3 and Cur exhibited increased cytotoxic activity to MDA-MB-231 cells. Therefore, the IC50 dose of FCANPs was calculated to be 3.4 µg/mL, which was approximately 2.38 times and 1.52 times lower than that of free Cur (7.9 µg/mL) and CNPs (5.3 µg/mL) based on Cur, respectively (Fig. 3B). Additionally, the apoptosis effects of various formulations were quantitatively investigated by flow cytometry analysis with the 2 μg/mL dose equivalent of Cur and 100 nM dose equivalent of antago3. As shown in Fig. 3C and Additional file 1: Fig. S3, the percentages of apoptotic MDA-MB-231 cells treated with free Cur, CNPs and ANPs were 11.4%, 14.5% and 12.1%, respectively. Significantly, the highest percentage of cell apoptosis (34.6%) was obtained in FCANPs-treated cells, demonstrating that co-delivery of antago3 and Cur into MDA-MB-231 cells could promote the cell apoptosis rate.

In TNBC patients, metastasis is the primary cause leading to high death rate. Therefore, developing novel and efficient methods to suppress metastasis is urgently needed and challenging. Targeting well-defined metastasis-related lncRNA has been recognized as novel anti-metastasis strategy [64]. LncRNA ASBEL has been previously described as a metastasis-promoting lncRNA and knockdown its expression could efficiently inhibit TNBC metastasis. Cur has also shown anti-metastasis activity in MDA-MB-231 cells and in animal model of TNBC [65]. Therefore, we were interested in investigating the regulatory effects of antago3 and Cur co-loaded FCANPs on migration and invasion of MDA-MB-231 cells. Two equivalent concentrations of Cur at 2.0 or 8.0 µg/mL (free Cur and CNPs) were employed in the study of transwell invasion assay. As observed in Fig. 3D, the number of invaded cells was obviously reduced after the treatment with 8 µg/mL of Cur and CNPs, while obvious cells migration was observed in cells treated with 2 µg/mL of Cur and CNPs compared with the control and BNPs-treated group. The total cell number was also measured. As presented in Additional file 1: Fig. S4, the total number of 2 µg of Cur and CNPs-treated cells remained up to 90%, while 8 µg/mL of Cur and CNPs resulted in a greater inhibitory effect of cell viability to nearly 50%. These data indicated that high concentration of Cur would inhibit both proliferation and invasion of MDA-MB-231 cells, while low concentration of Cur showed limited effect on MDA-MB-231 cells invasion. Furthermore, ANPs-treated MDA-MB-231 cells exhibited inhibition efficiency of migration to some extent. Most notably, FCANPs remarkably inhibited cell invasion to the maximum extent compared to any other single group. In addition, similar results were also reflected in the wound-healing assays. As seen in Fig. 3E, saline- and Cur-treated cells almost closed the wound within 48 h of incubation. CNPs and ANPs-treated cells showed a degree of migration inhibition. However, FCANPs inhibited cell migration more effectively (Additional file 1: Fig. S5). The results of the cell migration and invasion assay were consistent with the cytotoxicity assay results. Bcl-2 protein is a key apoptosis-regulating protein in various cancers and it has been considered as a conventional target gene for evaluating therapeutic effect [66]. Numerous researches have revealed the mechanisms through which c-Met signal pathway contributes to the migratory and invasive behaviors of TNBC [67]. Therefore, the expression of Bcl-2 and c-Met in MDA-MB-231 cells after different treatments were quantitatively examined. Downregulation of Bcl-2 and c-Met were found both in Cur, CNPs, ANPs and FCANPs-treated MDA-MB-231 cells at mRNA level (Fig. 3F, G) by qRT-PCR analysis and protein level by western-blot analysis (Fig. 3H). Expectedly, FCANPs exhibited a significant decrease in the expression of Bcl-2 and c-Met compared with other groups. Altogether, these results clearly demonstrated that FCANPs treatment showed the highest anti-cancer effects in MDA-MB-231 cells and effectively regulated relevant signaling pathways. As Wnt/β-catenin pathway induces tumor-suppressive effects through crosstalk of Bcl-2 and c-Met signal pathway. Both antago3 and Curcumin could inhibt Wnt/β-catenin signaling. This phenomenon may be caused by synergetic effects of antago3 and Curcumin through Wnt/β-catenin signaling [68].

### In vivo imaging and biodistribution analysis

**Fig. 4:**
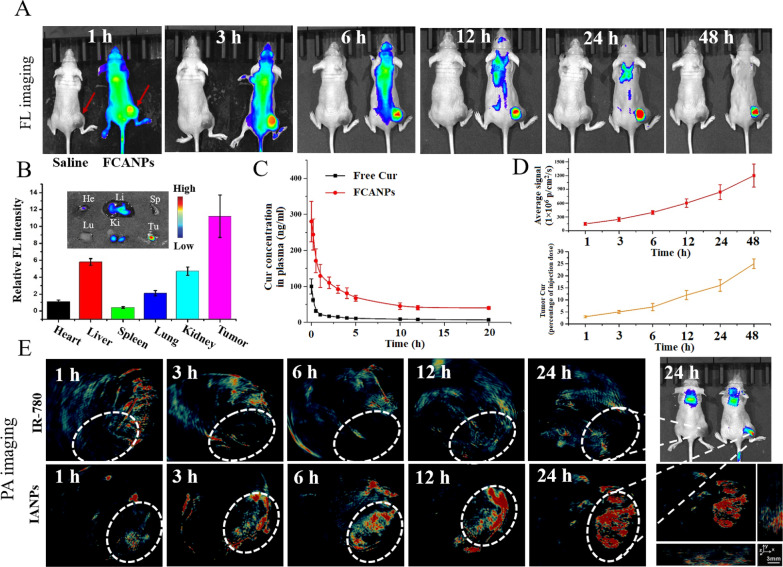
**A** In vivo NIR fluorescence imaging of mice after intravenous administration of saline and FCANPs for 1, 3, 6, 12, 24 and 48 h. Red arrows indicated the tumor site. **B** Ex vivo NIR fluorescence imaging of major organs (He: heart; Li: liver; Sp: spleen; Lu: lung; Ki: kidney; and Tu: tumors collected at 48 h post-injection of FCANPs and relative mean fluorescence intensity as calculated by semi-quantitative analysis. **C** Pharmacokinetics analysis of free Cur and FCANPs after intravenous injection (10 mg/kg of Cur) into back MDA-MB-231 tumor-bearing mice. The graph showed plasma concentration of Cur at different times post-injection (n = 3). **D** The concentration of Cur and relative fluorescence intensity of Cy-5.5 in tumor sites on different time points were plotted (n = 3). **E** In vivo PA images of mice taken at 1 h, 3 h, 6, 12 and 24 h after intravenous injection of free IR780 and IR780-loaded IANPs. The tumor site was visually indicated by the white circles

Prior to evaluating the in vivo anticancer activities, we next evaluated the in vivo fluorescence imaging to detect the biodistribution and the pharmacokinetic profile of intravenously administrated FCANPs in the tumor-bearing mice (≈ 200 mm^3^) at different time points. As presented in Fig. 4A, Cy-5.5 fluorescence was widely distributed through the body at the early stage after injection. Subsequently, strong fluorescence signal gradually accumulated in the tumor site could be observed. Obviously, the fluorescence signal increased dramatically after 3 h injection and reached a plateau at 24 h after injection. Significantly, strong and only fluorescence signals in the tumor site could be discerned after 48 h post-injection. At 48 h post-injection, major organs (heart, liver, spleen, lung, and kidney) and tumors were excised from the body and used for ex vivo fluorescence imaging (Fig. 4B). Consistent with the results from the above whole body NIR fluorescence imaging, high fluorescence intensity was detected in the tumors and low fluorescence signal was found in other organs. Moreover, the frozen tumor sections of tumors further confirmed the high accumulation of FCANPs in the tumor site and efficient cellular uptake of FCANPs by cancer cells (Additional file 1: Fig. S6). In the following, we investigated the in vivo pharmacokinetic (PK) behavior of free Cur and FCANPs by systematically injecting Cur and FCANPs into back tumor-bearing mice and calculated the concentration of Cur in plasma at different time points. As shown in Fig. 4C, the Cur concentration in the blood after injection of free Cur decreased rapidly, while the FCANPs exhibited prolonged circulation in the blood. We also evaluated the tumor accumulation of Cur at different time points post-injection. The results revealed that tumors exhibited increased Cur dose with extended time. Interestingly, we found that the increase of fluorescence intensity of Cy-5.5 in the tumor site from 1 to 48 h and exhibited the same tendency as the Cur content in the tumors (Fig. 4D). These results implied that the Cur delivery efficiency in the tumor site could be indirectly monitored by measuring the fluorescence of Cy-5.5 in the formulation of FCANPs.

To further directly elucidate that our established platform could deliver loaded drugs into tumor sites, IR780 iodide loaded nanocomplexes (IANPs) were also prepared in a similar way and used for both in vivo NIR fluorescence and PA imaging to directly visualize the biodistribution of loaded hydrophobic drug in the nanocomplexes. Hydrophobic IR780 iodide has been widely used as contrast agent for both NIR fluorescence imaging and photoacoustic (PA) imaging. As shown in Fig. 4E, the PA signal intensity tendency throughout the body was consistent with the above fluorescence imaging results, exhibiting gradually increase with extended time and the maximum accumulation at 24 h after intravenous injection of IANPs. At 24 h post-injection, the tumor site could be clearly visualized after the injection of IANPs for both PA and NIR fluorescence imaging modalities. In sharp contrast, free IR780 injected mice showed low or nearly no fluorescence and PA signal in the tumor site. Corresponding PA intensity of IR780 and IANPs in tumor sites at different time points were also shown (Additional file 1: Fig. S7). The results clearly demonstrated the time-dependent accumulation of IANPs at the tumor site, while free IR780 had few accumulations in the tumor site. As importantly, the fluorescence and PA imaging performance of IANPs clearly confirmed that CS-HA based nanocomplexes could efficiently deliver loaded hydrophobic drugs into tumors after systemic injection. Therefore, we could track and monitor the Cur/antago3 co-delivery route and efficiency through systemic injection patterns with the intrinsic fluorescence properties of FCANPs. All these results demonstrated that FCANPs had high tumor accumulation after intravenous injection and could deliver loaded drugs and genes into the tumor sites efficiently. The tumor targeting ability of FCANPs could be ascribed to the enhanced permeability and retention (EPR) effects-mediated passive targeting ability and HA-mediated active targeting ability.

### In vivo synergistic anti-tumor efficiency in different tumor models

**Fig. 5:**
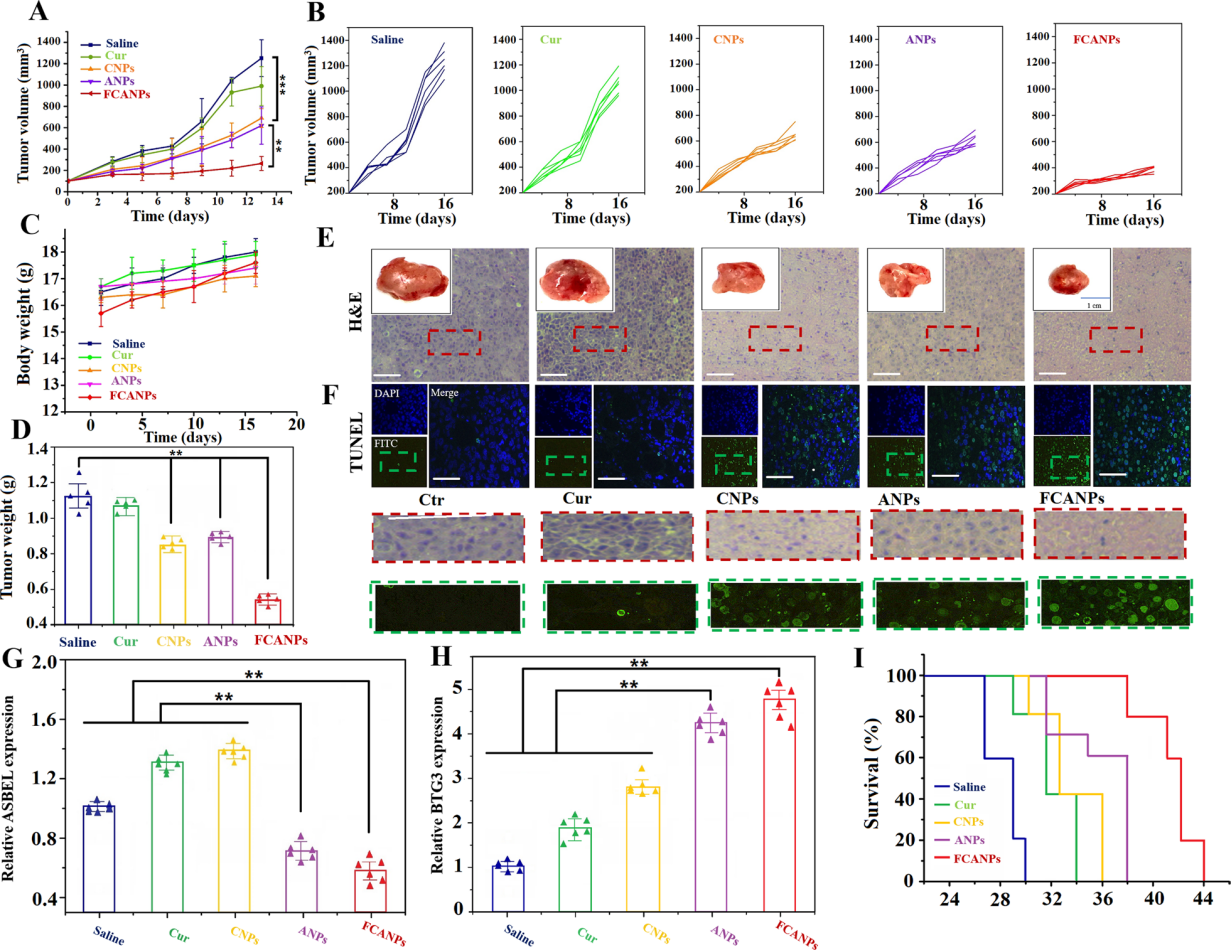
In vivo antitumor activities in back MDA-MB-231 tumor-bearing nude mice. **A** Growth curves of tumors in mice treated with saline, free Cur, CNPs, ANPs, and FCANPs (*n* = 5, mean ± SD, **p < 0.01). **B** Individual tumor growth curves of the mice in different groups. **C** Average body weights of mice during the treatment period. **D** Tumors were collected and weighed at the end of therapeutic experiments. **E**, **F** H&E and TUNEL staining of tumor sections after various treatments. The areas marked with dotted red and green rectangles are enlarged to reveal the changes of H&E and TUNEL pathological structure, respectively. Scale bar: 100 µm. **G**, **H** Relative lncRNA ASBEL and BTG3 expression in tumors by qRT-PCR analysis after different formulations treatment. **I** Survival rates of mice treated with various formulations. Data are shown as mean ± SD, n = 6. Statistical significance was calculated by one-way analysis of variance (ANOVA). *p < 0.05, **p < 0.01, ***p < 0.001

Based on the preferential in vitro collaborative biological and anti-cancer effects, and in vivo targeting accumulation and penetration of the FCANPs in tumors, we then further investigated the in vivo antitumor activity using the established subcutaneous back MDA-MB-231 tumor-bearing model of TNBC. Mice were randomly divided into five groups and received six injections of saline, free Cur, CNPs, ANPs and FCANPs at equivalent dose of Cur (10 mg/kg) and antago3 (1 mg/kg) when tumors reached approximately 100 mm^3^. The progressive growth curves of the xenograft tumors were summarized in Fig. 5A, B. It demonstrated that the tumors in saline-treated mice grew rapidly and could reach around 1300 mm^3^. Systemic injection of free Cur exhibited a very limited therapy result on tumor growth inhibition (900 mm^3^) in vivo was found compared with that of the saline group, which could be ascribed to the poor bioavailability and tumor accumulation of free Cur. The mice treated with single drug formulation of CNPs and ANPs had a mean tumor volume of ~ 650 mm^3^ and ~ 600 mm^3^, respectively, exhibiting moderate inhibition effects on tumor growth. In contrast, the FCANPs-treated mice showed a significantly greater tumor inhibition efficacy, indicating a joint antitumor effect of the collaborative lncRNA ASBEL knockdown and Cur delivery. Throughout the therapeutic experiment period, no significant change in the body weight of mice in all groups (Fig. 5C). Consistent with the tumor growth curves, the lowest weights of the tumors were found in the FCANPs-treated group (Fig. 5D).

We therefore performed H&E and TUNEL staining to evaluate the therapeutic effects. Compared to other groups, CNPs and ANPs treatments resulted in certain necrosis in the tumor cells by H&E analysis. Moreover, the FCANPs treatments showed significantly higher cell necrosis and apoptosis rates (Fig. 5E). TUNEL assay also showed significantly improved apoptotic effects by the combination of antago3 and Cur via FCANPs treatment, with the apoptotic rate reaching nearly 70%, indicating more efficient tumor cells killing effect mediated by FCANPs (Fig. 5F and Additional file 1: Fig. S8). In addition, effective downregulation of lncRNA ASBEL (Fig. 5G) and upregulation of BTG3 expression (Fig. 5H) were observed in ANPs and CANPs-treated tumors, suggesting an effective antag3 delivery and lncRNA ASBEL knockdown by ANPs and FCANPs. Significantly, the combination of antago3 and Cur by FCANPs significantly improved the survival of tumor-bearing mice relative to the other groups (Fig. 5I).

**Fig. 6:**
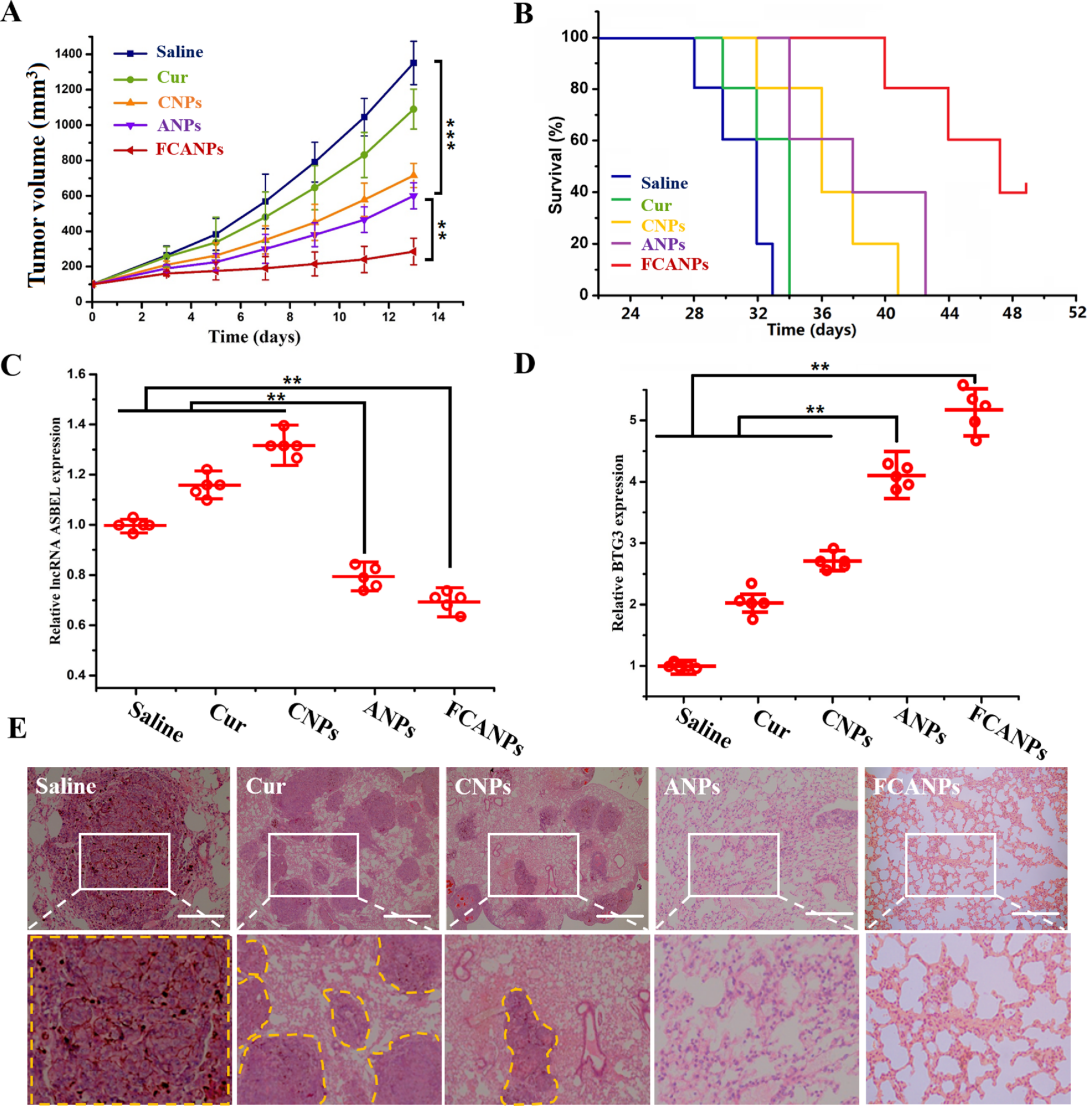
**A** In vivo antitumor activities in orthotopic MDA-MB-231 tumor-bearing nude mice. Tumor growth curves of mice injected with saline, Cur, CNPs, ANPs and FCANPs. **B** Survival rates of mice treated with various formulations. **C**, **D** Relative lncRNA ASBEL and BTG3 expression in tumors by qRT-PCR analysis after treatment with different formulations. **E** Representative H&E staining of lung sections from mice intravenously injected with MDA-MB-231 cells and received different treatments with saline, Cur, CNPs, ANPs and FCANPs. Scale bars, 100 µm. Data are shown as mean ± SD, n = 5. Statistical significance was calculated by one-way analysis of variance (ANOVA). **p < 0.01, ***p < 0.001

Furthermore, we also investigated the therapeutic efficiency of FCANPs in MDA-MB-231 breast-tumor xenografts grown orthotopically in the mammary fat pads of the BALB/c nude mice model. As shown in Fig. 6A, similar to the above antitumor activities in the back MDA-MB-231 tumor-bearing mice model, FCANPs treatment exhibited the highest tumor inhibition effects during the whole therapeutic period up to 14 d. After the treatment, the tumor-bearing mice were killed and the tumors were collected, photographed and weighed. The average tumor weight in the FCANPs-treated mice was the lowest. Similarly, FCANPs treatment significantly extended the survival time of mice compared with other single drugs of CNPs and ANPs and control saline groups (Fig. 6B). Furthermore, the expressions of lncRNA ASBEL and BTG3 in tumors were consistent with the above results in the back MDA-MB-231 tumor-bearing mice (Fig. 6C, D). Lethal pulmonary are the main cause of mortality in breast cancer patients, especially in TNBC patients. In vitro studies have demonstrated that the ANPs and FCANPs formulations containing antago3 could inhibit cell migration/invasion effectively. Therefore, we investigated the anti-metastasis efficiency of various treatments in vivo conditions. The lungs were evaluated microscopically followed by H&E staining. As shown in Fig. 6E, metastatic tumor cells occupied the lung of saline- and free Cur-treated mice. In comparison, the amounts of metastatic foci in the lung were markedly inhibited by ANPs and FCANPs treatment, indicating those treatments containing antago3 were capable of preventing tumor spread.

### In vivo biocompatibility and biosafety studies

**Fig. 7:**
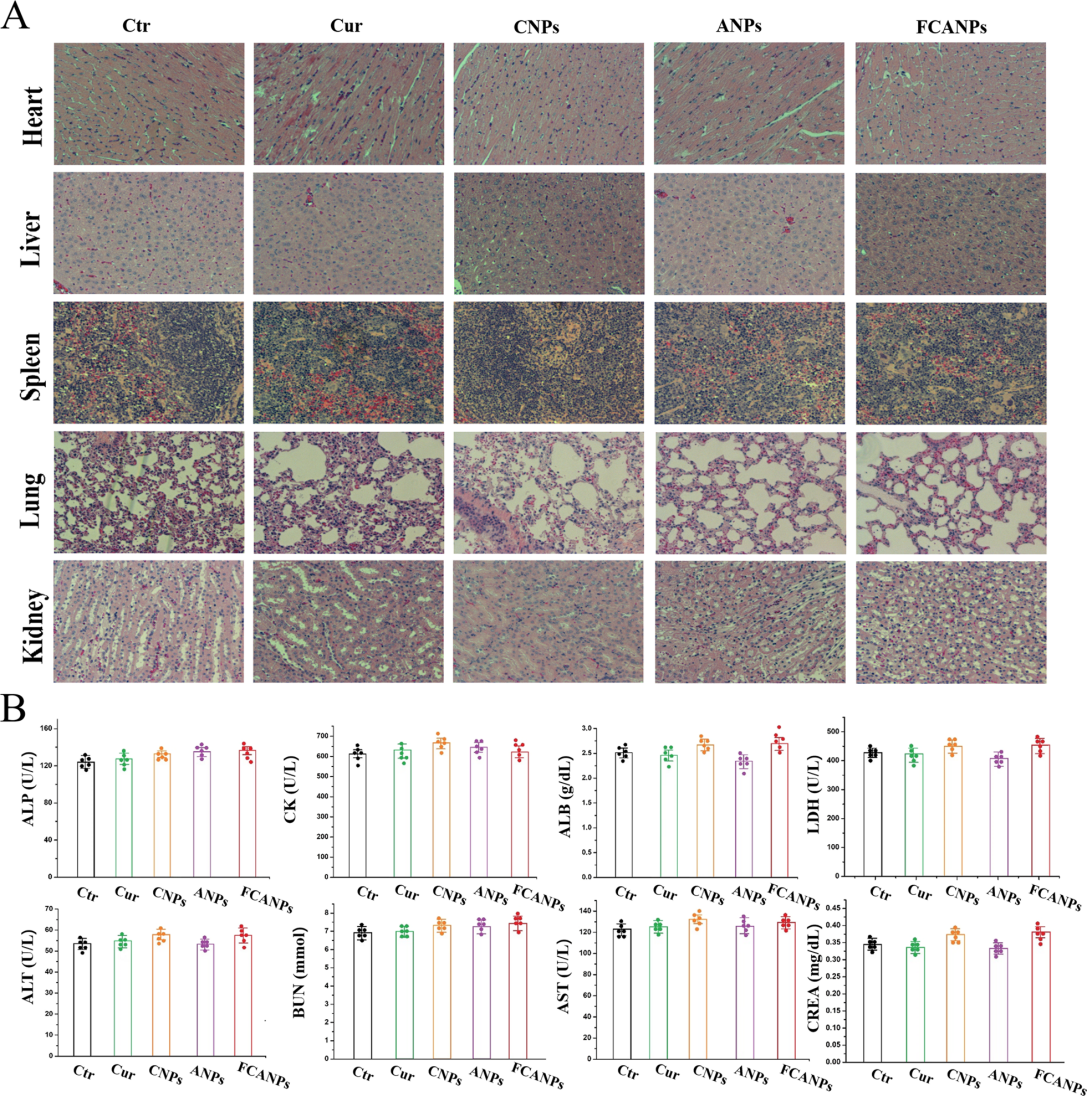
Biocompatibility and safety evaluation. **A** Representative H&E staining sections of heart, liver, spleen, lung and kidney collected from mice in different groups (Ctr, free Cur, CNPs, ANPs and FCANPs) at 16 days post-treatment. **B** Serologic and biochemical analysis. The level of ALP, ALB, ALT, AST, CK, LDH, BUN and CREA. Data are presented as the mean ± SD (n = 5)

In vivo biocompatibility and safety of the applied FCANPs formulation was the prerequisite and very important factor for further application. We next assessed the potential in vivo toxicity of FCANPs via H&E analysis, serologic and biochemical analysis. First of all, major organs including heart, liver, spleen, lung, and kidney were collected and examined by H&E staining at the end of therapeutic period in the back tumor-bearing mice to verify the biocompatibility of FCANPs. As shown in Fig. 7A, neither noticeable apoptosis nor obvious pathological tissue damage was observed in these organs in all groups, demonstrating that various formulations of treatments had limited side effects to normal organs. Additionally, serologic and biochemical analysis associated with liver function, myocardial enzyme spectrum, and renal function was also conducted. As depicted in Fig. 7B, the liver function indexes [alanine aminotransferase (ALT)/aspartate aminotransferase (AST)/alkaline phosphatase (ALP)/albumin (ALB)], heart function indexes [creatine kinase (CK)/lactate dehydrogenase (LDH)] and renal function indexes [blood urea nitrogen (BUN)/creatinine (CREA)] were all maintained within normal range and no additional serum biochemical abnormality was observed in mice treated with various formulations, indicating good biocompatibility of FCANPs on the tumor-bearing mice. On the whole, the above results illustrated that the satisfying biocompatibility and safety of FCANPs system for the effective treatment of TNBC.

## Conclusions

In summary, we have successfully developed FCANPs polyelectrolyte nanocomplexes based on HA and CS assemblies for the co-delivery of anti-lncRNA ASBEL antago3 and Cur, which could achieve synergetic treatment efficiency against TNBC. NIRF Cy-5.5 modification on the surface of FCANPs could allow for synchronous tumor imaging and therapy monitoring. FCANPs-mediated codelivery of antago3 and Cur exhibited synergetic anti-cancer effects, which efficiently inhibited cell proliferation, migration/invasion as well as induced apoptosis. After treatment with FCANPs, effective down-regulation of lncRNA ASBEL and synchronous regulation of BTG3, Bcl-2 and c-Met genes could be observed. Through systemic injection of FCANPs, targeted and preferential accumulation of the FCANPs around the tumor tissue could be clearly distinguished by the NIR fluorescence imaging. FCANPs-mediated antago3 and Cur co-delivery significantly suppressed the growth of MDA-MB-231-derived back and orthotopic tumor xenografts, extended animal survival and inhibited lung metastasis with good biocompatibility in nude mice. Collectively, our findings demonstrated that collaborative oncogenic LncRNA ASBEL knockdown and Cur delivery with FCANPs presented a promising approach for TNBC theranostics.

## Materials and methods

### Materials

Curcumin (Cur) and sodium tripolyphosphate (TPP) were purchased from Aladdin Co. LTD (Beijing, China). Sodium hyaluronate (HA, MW = 170 kDa) was provided by Dongyuan Biochem. Co. Ltd. (Jiangsu, China). Chitosan hydrochloride (CS, MW = 110 kDa) was provided from Zhejiang Aoxing Biochem Co. Ltd. Cell counting kit-8 was purchased from Dojindo Laboratories (Tokyo, Japan). Ethylenediamine, 1-ethyl-3(3-dimethylaminopropyl) carbodiimide (EDC), *N*-hydroxysuccinimide (NHS), penicillin and streptomycin, dialysis membrane (cutoff 3000 Da) were provided by Sigma-Aldrich (St. Louis, MO, USA). Cyanine-5.5 amine (Cy-5.5-NH_2_) was purchased from Xi’an Ruixi Biological Technology Co., LTD. Antago3 with or without Cyanine-3 (Cy-3) labeling (antago3, 5′-CGGTCTGGGCCGCCGA-3′) and the PCR primers were synthesized and supplied by Sangon Biotech Co. LTD (Shanghai, China). DNase I was purchased from Beyotime Biotech Co. LTD (Shanghai, China). Dulbecco’s modified Eagle’s medium (DMEM), penicillin, streptomycin, and fetal bovine serum (FBS) were purchased from Gibco BRL (Grand Island, NY, USA). Antibodies were purchased from Abcam, Inc. All the other agents were analytical-grade and used as received unless otherwise specified. Triple distilled water was used throughout the experiments and was supplied by a Milli-Q purification system (Millipore Co., Billerica, MA, USA).

### Preparation of BNPs, CNPs, CANPs and FCANPs

According to the protocols described in our previous study related to the preparation of HA-CS-based SPEC NPs [55], in this study, we prepared blank NPs (BNPs), Cur-loaded NPs (CNPs), antago3-loaded NPs (ANPs) and Cur-antago3-loaded NPs (CANPs) with slight modifications, respectively. Briefly, fresh stock solutions of HA (1.25 mg/mL), CS (0.6125 mg/mL) and TPP (0.5 mg/mL) were prepared in DNase-free water followed by sonication for 10 min. All the solutions were filtered through a 0.22 µm pore size filter to remove any macroscopic material and bacteria possibly present. 100 µL of TPP solution (0.5 mg/mL) was firstly mixed with 2 mL of HA solution (1.25 mg/mL). 2 mL of CS solution (0.6125 mg/mL) was dropwise added to the HA-TPP mixed solution. After the addition, the complexation was carried at room temperature and under magnetic stirring for a duration of 10 min. The solution was then centrifuged for 10 min with the speed of 3000×*g* in a glycerol bed. The resulting precipitate was then washed twice and dispersed in DNase-free water as BNPs solution for further analysis and use.

The same procedure was followed as described for the preparation of CNPs, ANPs and CANPs. For the preparation of CNPs, various amounts of Cur dissolved in methanol was firstly added to 2.0 mL of HA-TPP solution phase. Then 2 mL of CS solution (0.6125 mg/mL) was dropwise added to the Cur-HA-TPP mixed solution. For the preparation of ANPs, various amounts of antago3 dissolved in 100 µL DNase-free water were first added to TPP-HA solution phase. Then 2 mL of CS solution (0.6125 mg/mL) was dropwise added to the antago3-HA-TPP mixed solution. For the preparation of CANPs, various amounts of Cur dissolved in methanol and antago3 dissolved in DNase-free water were simultaneously added to the TPP-HA solution. Then 2 mL of CS solution (0.6125 mg/mL) was dropwise added to the Cur- antago3-HA-TPP mixed solution.

For preparation of NIR fluorescent CANPs (FCANPs), CANPs were therefore surface decorated with NIR dye Cy-5.5. In brief, CANPs were chemically modified with NH_2_-modified Cy-5.5 (Cy-5.5-HN_2_) (1 mg/mL) in the presence of EDC (5 mg/mL) and NHS (5 mg/mL) for 2 h reaction. The mass ratio of CANPs to Cy-5.5-NH_2_ was fixed at 20:1. To remove unconjugated free Cy-5.5-NH_2_, the FCANPs solution was centrifuged using the ultrafiltration (Molecule cut-off, 3000 Da MWCO) for 30 min with the speed of 3000×*g* in a glycerol bed. After purification, the FCANPs were redispersed and used for further experiments. The conjugation efficiency of Cy-5.5-NH2 was 56.7% by quantifying the fluorescence intensity of Cy-5.5.

### Characterization

Dynamic light scattering (DLS) was used to determine the hydrodynamic size and zeta potential with a Malvern ZetaSizer Nano-ZS (Malvern Instruments Ltd., Malvern, UK) at 25 °C. The morphologies of BNPs, CNPs, ANPs and CANPs were observed by transmission electron microscopy (TEM) (Tecnai G2 20 S-TWIN, FEI Company, Philips, Netherlands). The samples on carbon-coated copper grid were negatively stained with 0.3% w/v of uranyl acetate. Fluorescence spectra was recorded on a LS-55 fluorescence spectrometer (PerkinElmer, Fremont, CA). Fluorescence spectra of Cy-5.5 were obtained using an excitation wavelength of 675 nm. NIR Fluorescence images were obtained using the CRI Maestro Imaging System (Cambridge Research and Instrumentation Inc., USA). The encapsulation efficiency (EE%) and loading content (LC%) of Cy-3-antago3 and Cur were detected by quantifying the fluorescence intensity of Cur and Cy-3 in the supernatant after preparation of CANPs.

### Agarose gel electrophoresis

We used the gel retardation assay to evaluate the DNase I stability of naked antago3 and antago3 loaded in CANPs. The stability of anago3 in CANPs was measured using the sodium dodecyl sulfonate (SDS) displacement assay. Both naked antago3 and CANPs solutions were incubated with 0.5 U DNase I at 37 °C for different time points. At predetermined time point, 10 µL of the mixtures were taken out. 5 µL of 2% SDS and 5 µL of 10% glycerine were added to the above mixed solutions. Then, the samples were loaded onto 2% agarose gel in tris-acetate-ethylenediaminetetraacetic acid (EDTA) buffer containing 0.5 μg/mL of GelRED TM (Biotium, USA). Electrophoresis was performed at 110 V for 10 min and the gel was visualized by with the Bio-Rad imaging system.

### In vitro Cur release profiles

The in vitro release profile for Cur from FCANPs was investigated in different pH sink conditions using the dialysis diffusion method. A 2 mL dispersion of CANPs was added to a dialysis bag (Cutoff 3000 Da) and then dialyzed against 30 mL of different PBS solutions (pH 7.4 and 5.5) containing 1% Tween-80 under constant horizontal shaking (100 rpm). At different time intervals, 1 mL of the solution was collected and equal volume of PBS was complemented again. The amount of released Cur was evaluated by high-performance liquid chromatography (HPLC) method. The release experiments were repeated in triplicate.

### Cellular uptake study

Human triple-negative breast cancer cells (MDA-MB-231) were cultured in DMEM medium replenished with 10% FBS, 1% penicillin and 1% streptomycin at 37 °C in a humidified atmosphere containing 5% CO_2_. MDA-MB-231 cells were seeded in the glass-bottom dish at the density of 1 × 10^5^ cells per dish and incubated overnight. Then, 50 µg/mL FBNPs and 50 µg/mL FCANPs were added into the dishes and incubated for 4 h. For HA block studies, the cells were pre-incubated with medium containing 10 mg/mL of HA for 1 h. Then, the medium was replaced by fresh medium containing 50 µg/mL FBNPs or 50 µg/mL FCANPs and incubated for another 4 h. Finally, the cells were washed three times with PBS and then observed using confocal microscopy (CLSM, Carl Zeiss, Boston, MA). In the experiment of FBNPs-treated cells, the nuclei were stained with DAPI for 15 min. The intracellular localization of Cur was observed by excitation wavelength at 488 nm laser and the emission wavelength was 530 nm. The excitation and emission wavelength of Cy-3 were 550 nm and 570 nm, respectively. The Cy-5.5 fluorescence was detected under 675 nm laser excitation and the emission was collected under 694 nm.

### In vitro cytotoxicity analysis

The cytotoxicity of BNPs in MDA-MB-231 cancer cells and human umbilical vein endothelial cells (HUVEC) were tested by the CCK-8 assays. Briefly, the cells were seeded in 96-well plates with a density of 5 × 10^3^ cells per well for 24 h. Then, the medium was replaced with fresh medium containing different concentrations of BNPs (0, 10, 20, 50, 100, 200, 400 and 800 µg/mL) and incubated for additional 24 h. Subsequently, the cells were incubated with free serum-free medium with 10% CCK-8 kit for 30 min at 37 °C. Finally, the absorbance was detected by microplate reader (Bio-Rad 550, USA) under 450 nm.

### Cell viability and apoptosis analysis

MDA-MB-231 were seeded in a 96-well plate at a density of 5 × 10^3^ per well in DMEM containing 10% FBS in a humidified atmosphere of 5% CO_2_. After being incubated for 24 h at 37 °C, the cells were treated with different formulations (saline, free Cur, CNPs, ANPs and FCANPs). The concentrations (free Cur, CNPs, FCANPs) were represented by the concentrations of Cur (0–24 µg/mL). The concentration of ANPs was represented by the concentrations of antago3 (100 nM/mL). The control cells were treated by saline. After 24 h incubation, the cell viabilities were measured by CCK-8 assay as described above. The IC 50 was determined by nonlinear regression analysis using the equation for a sigmoid plot. To detect cell apoptosis, MDA-MB-231 cells were collected at 24 h after different formulations treatment and apoptosis assay was measured by using an Alexa Fluor 488 annexin V/Dead Cell Apoptosis Kit (Invitrogen) with a FACSCalibur FCM (BD). The apoptosis effects of various formulations were quantitatively investigated by flow cytometry analysis with the 2 μg/mL dose equivalent of Cur and 100 nM dose equivalent of antago3. The concentration of BNPs was 50 µg/mL.

### Scratch wound healing and transwell migration assays

For the scratch wound healing assay, MDA-MB-231 cells (1 × 10^4^) were seeded on 24-well plates for 24 h. A single straight scratch was made in the monolayer with a 100 µL of pipet tip. After washing the detached cells with PBS, the cells were treated with different formulations (PBS, Cur, ANPs, CNPs, FCANPs) and monitored for up to 48 h. The concentrations (free Cur, CNPs, FCANPs) were represented by the concentrations of Cur (2 µg/mL). The concentration of ANPs was represented by the concentrations of antago3 (100 nM/mL). The distance of migration from the monolayer to the wounded area during this time period was measured. Cell migration was measured using cell transwell assay with a pore size of 8 µm. After different formulations treatments (PBS, Cur, ANPs, CNPs, FCANPs) for 24 h, 5 × 10^3^ MDA-MB-231 cells were plated on transwell inserts (VWR, Radnor, PA) coated with 0.28 mg/mL Corning™ Matrigel™ Membrane Matrix (Corning, NY). The concentrations (free Cur, CNPs, FCANPs) were represented by the concentrations of Cur (2 µg/mL). The concentration of ANPs was represented by the concentrations of antago3 (100 nM/mL). 10% of formalin was used to fix the migrated cells for 10 min. After washing with PBS three times, the migrated cells were stained with 0.05% crystal violet. The inserts were washed under running water to remove excess stain and set to dry overnight. Then, the invaded cells were viewed by the microscope. The migration ratios were counted using the ImageJ software.

### Quantitative real-time PCR and western-blot analysis

The quantitative real-time PCR (qRT-PCR) experiments were carried out to detect the expression level of lncRNA ASBEL and mRNA expression levels of BTG3, c-Met and Bcl-2 as previously described [27]. All RNAs were normalized to the expression level of GAPDH. Western-blot assay was also used to detect the protein expressions of BTG3, c-Met and Bcl-2. Total proteins including cells and tumor tissues were extracted as previously described [27]. Protein extracts were separated by SDS-PAGE, transferred onto PVDF membranes and immunoblotted with primary antibodies overnight. After three washes, the membranes were incubated with secondary antibodies at room temperature. After being washed with TBST, the protein signals were detected using the ChemiDocTM XRS + Imaging System (BioRad), which was supplemented with 200 µL chemiluminescence ECL kit (Beyotime Biotechnology, Beijing, China) and visualized using.

### Animal model

All animal studies were performed in accordance with the Guidelines for Care and Use of Laboratory Animals of National Center for Nanoscience and Technology and approved by the Institutional Animal Care and Use Committee (IACUC) in compliance with Chinese law for experimental animals. Female BALB/c-nude mice (Beijing Weitong Lihua Experimental Animal Technical Co., Ltd.) were housed in an environmentally controlled animal facility with a regular 12/12 light/dark cycle.

### In vivo imaging, biodistribution and pharmacokinetics studies

For in vivo fluorescence imaging, tumor model was established. BALB/c-nude mice were injected subcutaneously with 5 × 10^6^ MDA-MB-231 cells suspended in 0.1 mL of PBS on the right flank. When the volume of subcutaneous tumors reached around 200 mm^3^, the mice were separated into two groups at random for various treatments (n = 3 per group). The mice were administrated with 0.1 mL of saline and FCANPs (10 mg/kg of Cur, 20 µg of Cy-5.5) through intravenous injection, respectively. Imaging was performed at predetermined time points (1 h, 3 h, 6 h, 12 h, 24 h and 48 h), the mice were anesthetized and scanned by the multispectral fluorescence imaging system (Cri-M2, CRI USA). After imaging, mice were sacrificed for the harvest of major organs (liver, lung, spleen, kidneys, and heart) for analyzing the ex vivo fluorescence distribution. The tumors were also frozen and cut into 10 µm thickness using a Leica cryostat, and imaged by fluorescence microscope.

For pharmacokinetics study, back MDA-MB-231 tumor-bearing mice were intravenously injected with 0.1 mL of FCANPs (10 mg/kg of Cur) and blood was collected at predetermined intervals. The blood was centrifuged at 10,000 rpm for 10 min to obtain plasma. 2 mL of methyl alcohol was added to 100 μL of plasma and vortexed for 3 min to extract Cur. The resulting solution was then centrifuged for 10 min, the supernatant was injected for HLPC analysis and the Cur concentration was measured. At 48 h post-injection, the intensity of the ex vivo fluorescence signal of the tumor site was semi-quantified with the multispectral fluorescence imaging system and the Cur concentration in the tumor tissue was also measured as described above.

Hydrophobic IR780 was loaded as IANPs and PA imaging was used to determine the biodistribution of IANPs. The tumor-bearing mice were intravenously injected with 100 μL of solution containing IANPs and free IR780. In vivo PA imaging at different time points after systemic injection was obtained with the multispectral optoacoustic tomography scanner (MSOT, iThera Medical).

### In vivo antitumor efficacy in different MDA-MB-231 tumor-bearing models

MDA-MB-231 tumor-bearing nude mice models were established as described above. When the volume of subcutaneous tumors reached around 100 mm^3^, the mice were separated into five groups at random for various treatments (n = 6 per group). The mice were intravenously injected with saline, free Cur (Cur dose: 10 mg/kg), CNPs (Cur dose: 10 mg/kg), ANPs (antago3 dose: 1 mg/kg) and CANPs (Cur dose: 10 mg/kg; antago3 dose: 1 mg/kg) every other day for a total of five doses. Mice were weighed and the tumor dimensions were recorded once every 3 days during the treatment period. The volumes of tumor were calculated according to the following equation: (length × width^2^)/2. These mice were sacrificed at 24 days post-injection, the tumors and major organs (liver, heart, lung, spleen, and kidney) were excised for hematoxylin–eosin (H&E) staining and terminal deoxynucleotidyl transferase dUTP nick end labeling (TUNEL) staining. The morphology of each section was observed by a fluorescence microscope (Olympus, Japan). For analysis of lncRNA ASBEL and BTG3 level in tumors, qRT-PCR measurements were performed. For analysis of mice survival rates, tumor-bearing mice were subjected to the corresponding treatment as above and the dates of mortality were recorded.

To establish the orthotopic TNBC mice model, a total of 5 × 10^6^ MDA-MB-231 cells mixed with an equal volume of Matrigel were subcutaneously injected into the right side of the fourth fat pad of nude mice. When the volume of subcutaneous tumors reached around 100 mm^3^, the mice were separated into five groups (n = 5 per group) and treated with different formulations as above. The body weights of mice and the volumes of tumor were calculated every 3 days. For analysis of mice survival rates, tumor-bearing mice were subjected to the corresponding treatment as above and the dates of mortality were recorded.

To investigate the anti-metastasis abilities, we built pulmonary metastasis model of MDA-MB-231 cancer through intravenous injection of 1 × 10^6^ MDA-MB-231 cells suspended in 0.1 mL of PBS. On day 7, the mice were separated into five groups (n = 5 per group) and treated with different formulations as above. After treatments, tumor metastases in lungs were observed by H&E analysis.

### Biochemistry index and histology analysis

Mice were sacrificed under anesthesia at the end of therapeutic period. Major organs (heart, liver, spleen, lung, and kidney) were collected and then fixed in paraformaldehyde, and embedded in paraffin and stained with H&E. The images of the tissues were observed by the fluorescence microscope. After that, mice blood samples were collected and the blood biochemistry analysis was measured by auto hematology analyzer.

### Statistical analysis

All the values were expressed as mean ± SD (stand deviation). A One-way ANOVA test was used to determine significance among different groups. *p < 0.05, **p < 0.01, ***p < 0.001 were considered statistically significant.

### Supplementary Information

**Additional file 1: Table S1.** Stability of CANPs based on the size and EE% of Cur during 2-weeks of storage. **Figure S1.** TEM images of BNPs, CNPs and ANPs. Scale bar: 200 nm. **Figure S2.** BSA adsorption assays on the FCANPs after different time co-incubation at 37 °C. **Figure S3.** Apoptotic cell numbers of MDA-MB-231 cells after treated with different formulations. The apoptotic cells included both early and late apoptotic cells. **Figure S4.** The total cells number was measured in the transwell invasion assays at equivalent dose of Cur (2 μg/mL and 8 μg/mL) Data are shown as mean ± SD, n = 3. Statistical significance was calculated by one-way analysis of variance (ANOVA). **p < 0.01. **Figure S5.** Quantitative evaluation on the percentage of the wound window closed after different treatments, values were normalized by the initial wound window width. Data are shown as mean ± SD, n = 3. Statistical significance was calculated by one-way analysis of variance (ANOVA). *p < 0.05, **p < 0.01, ***p < 0.001. **Figure S6.** Histological and fluorescent images of frozen tumor sections (10 µm thick). Cy-5.5 fluorescence signal was shown in red. **Figure S7.** Quantitative analysis of relative PA intensity of IR780 in xenograft tumors at indicated time points after intravenous administration of IANPs and free IR780. data are shown as mean ± SD, n = 3. Statistical significance was calculated by performed by paired Student’s t test. *p < 0.05. **Figure S8.** TUNEL-positive cells (%) in tumors of each group. data are shown as mean ± SD, n = 6. Statistical significance was calculated by one-way analysis of variance (ANOVA). **p < 0.01. **Figure S9.** Relative C-met and Bcl-2 expression after different treatments by western-blot analysis. Relative band intensity was normalized by GAPDH. Data are shown as mean ± SD, n = 3. Statistical significance was calculated by one-way analysis of variance (ANOVA). **p < 0.01, ****p < 0.0001.

## Data Availability

Without restrictions.

## Abbreviations

lncRNAs: Long non-coding RNAs
TNBC: Triple-negative breast cancer
HA: Hyaluronic acid
CS: Chitosan hydrochloride
NIRF: Near-infrared fluorescence
TPP: Tripolyphosphate
SPECs: Self-assembled polyelectrolyte nanocomplexes
PA: Photoacoustic
