# Supplementary material for: Coordinated modulation of long non-coding RNA ASBEL and curcumin co-delivery through multicomponent nanocomplexes for synchronous triple-negative breast cancer theranostics
Source: J Nanobiotechnology. 2023 Oct 31;21:397. doi: 10.1186/s12951-023-02168-8 (PMC10617238; doi:10.1186/s12951-023-02168-8)
Supplement: Supplementary file 1 — Additional file 1: Table S1. Stability of CANPs based on the size and EE% of Cur during 2-weeks of storage. Figure S1. TEM images of BNPs, CNPs and ANPs. Scale bar: 200 nm. Figure S2. BSA adsorption assays on the FCANPs after different time co-incubation at 37 °C. Figure S3. Apoptotic cell numbers of MDA-MB-231 cells after treated with different formulations. The apoptotic cells included both early and late apoptotic cells. Figure S4. The total cells number was measured in the transwell invasion assays at equivalent dose of Cur (2 μg/mL and 8 μg/mL) Data are shown as mean ± SD, n = 3. Statistical significance was calculated by one-way analysis of variance (ANOVA). **p < 0.01. Figure S5. Quantitative evaluation on the percentage of the wound window closed after different treatments, values were normalized by the initial wound window width. Data are shown as mean ± SD, n = 3. Statistical significance was calculated by one-way analysis of variance (ANOVA). *p < 0.05, **p < 0.01, ***p < 0.001. Figure S6. Histological and fluorescent images of frozen tumor sections (10 µm thick). Cy-5.5 fluorescence signal was shown in red. Figure S7. Quantitative analysis of relative PA intensity of IR780 in xenograft tumors at indicated time points after intravenous administration of IANPs and free IR780. data are shown as mean ± SD, n = 3. Statistical significance was calculated by performed by paired Student’s t test. *p < 0.05. Figure S8. TUNEL-positive cells (%) in tumors of each group. data are shown as mean ± SD, n = 6. Statistical significance was calculated by one-way analysis of variance (ANOVA). **p < 0.01. Figure S9. Relative C-met and Bcl-2 expression after different treatments by western-blot analysis. Relative band intensity was normalized by GAPDH. Data are shown as mean ± SD, n = 3. Statistical significance was calculated by one-way analysis of variance (ANOVA). **p < 0.01, ****p < 0.0001. [file 12951_2023_2168_MOESM1_ESM.doc]

**Additional file 1**

Table S1. Stability of CANPs based on the size and EE% of Cur during two-weeks of storage

|  | initial | 3 days | 7 days | 10 days | 14 days |
| --- | --- | --- | --- | --- | --- |
| Hydrodynamic size | 223.1 | 225.2 | 228.3 | 231.2 | 241.6 |
| EE% of Cur | 58.2 | 56.3 | 54.6 | 53.3 | 52.7 |


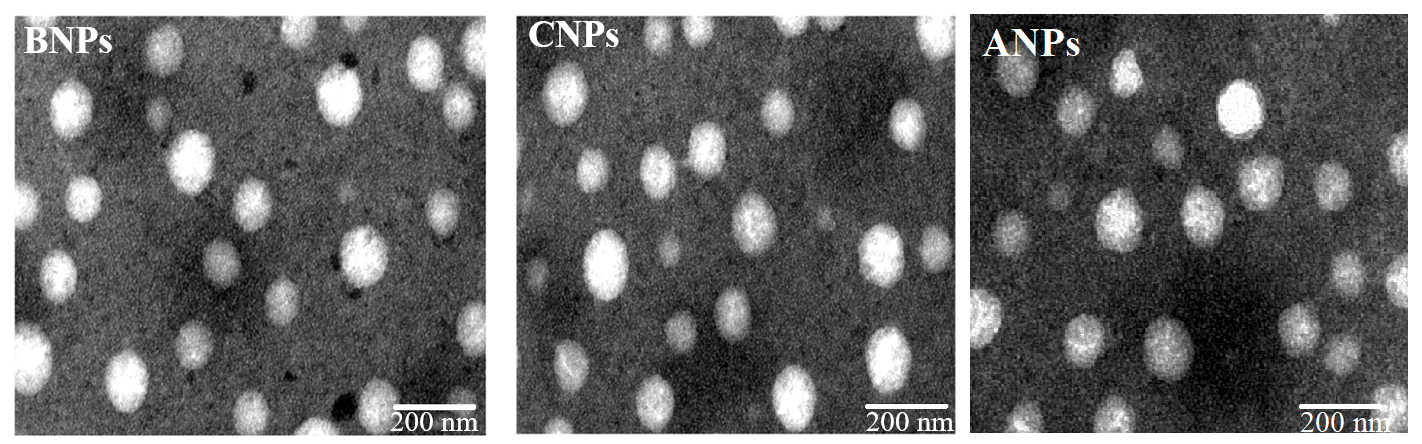


Figure S1. TEM images of BNPs, CNPs and ANPs. Scale bar: 200 nm


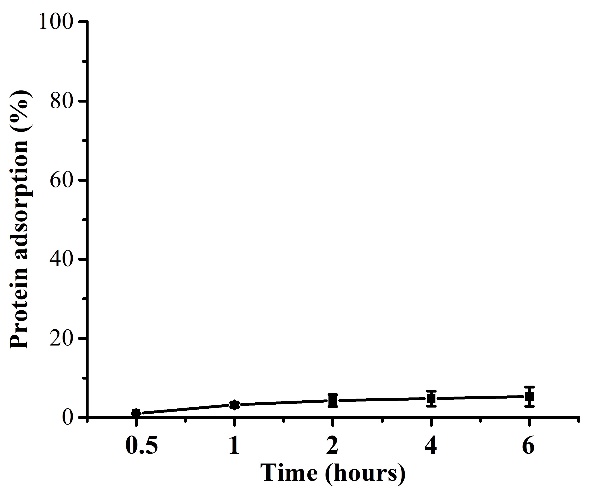


Figure S2. BSA adsorption assays on the FCANPs after different time co-incubation at 37 °C.


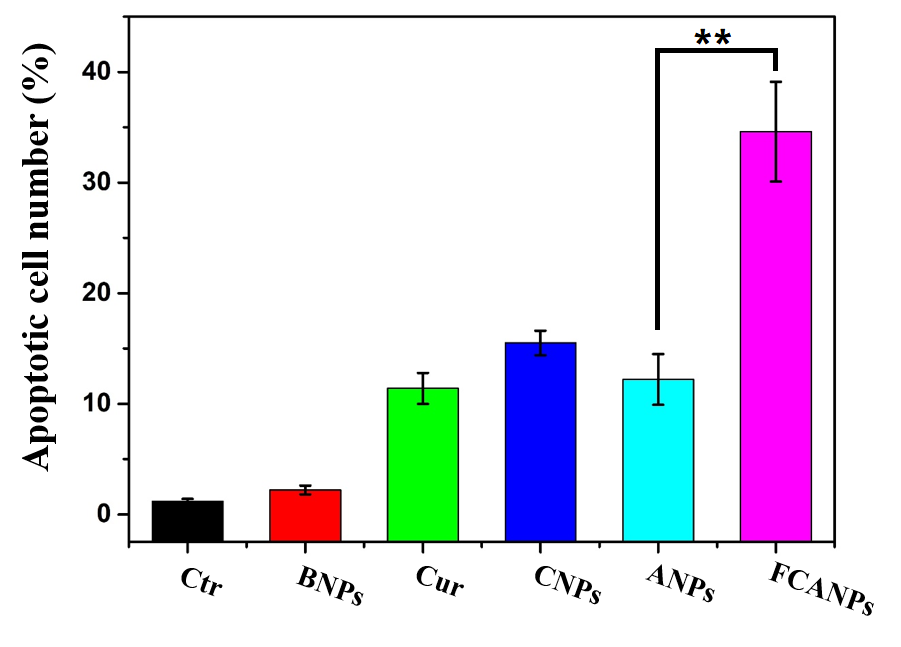


Figure S3. Apoptotic cell numbers of MDA-MB-231 cells after treated with different formulations. The apoptotic cells included both early and late apoptotic cells.


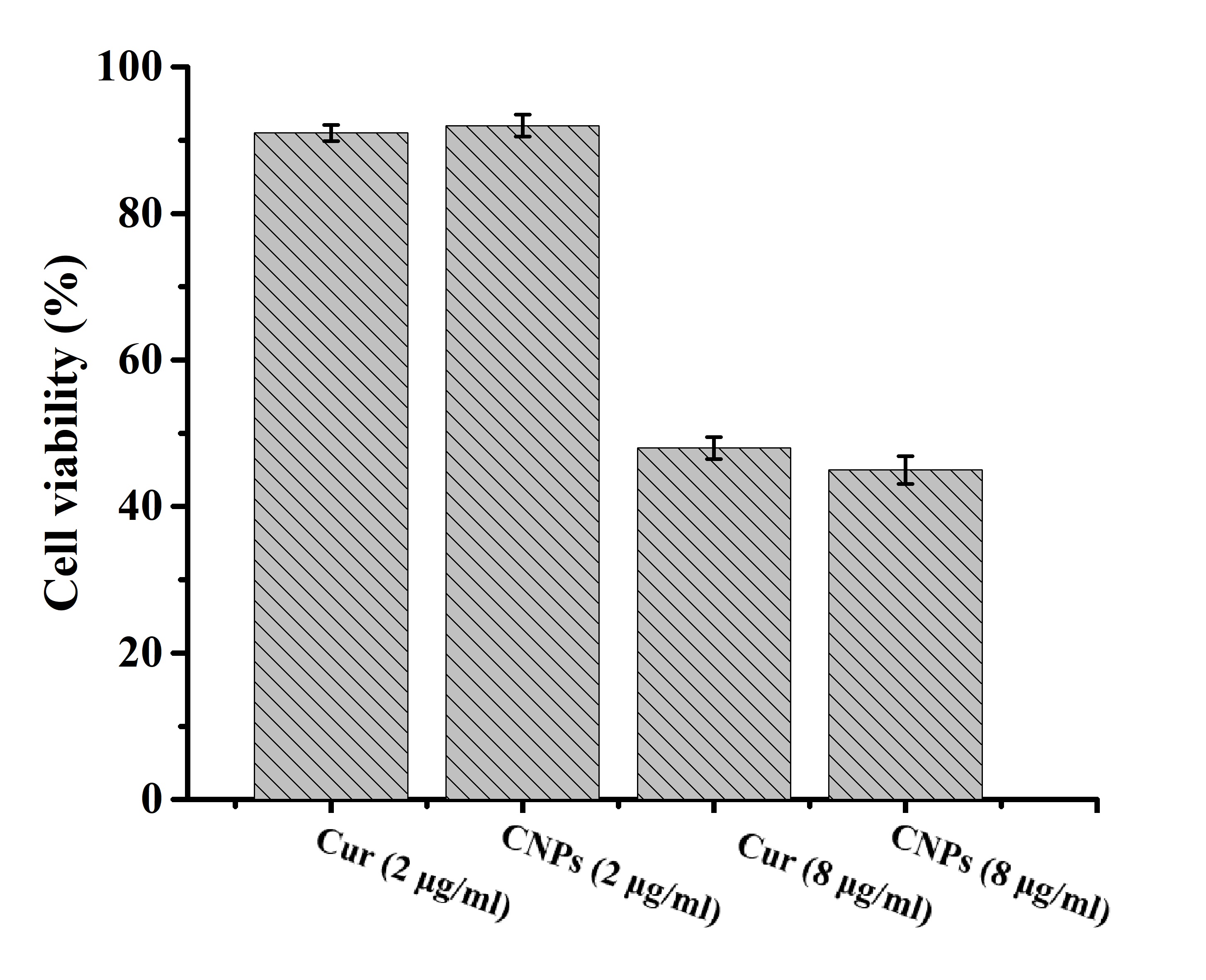


Figure S4. The total cells number was measured in the transwell invasion assays at equivalent dose of Cur (2 μg/ml and 8 μg/ml) Data are shown as mean ± SD, n = 3. Statistical significance was calculated by one-way analysis of variance (ANOVA). **p＜0.01.


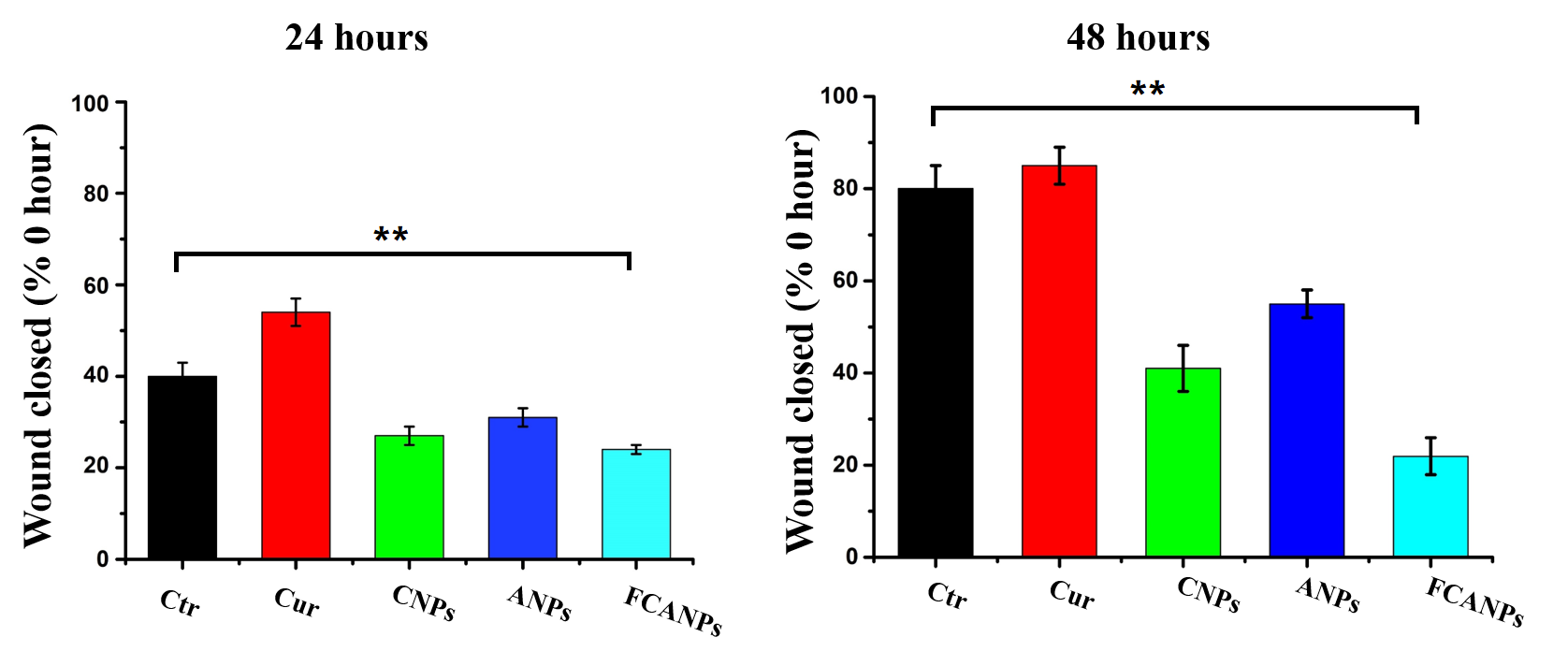


Figure S5. Quantitative evaluation on the percentage of the wound window closed after different treatments, values were normalized by the initial wound window width. Data are shown as mean ± SD, n = 3. Statistical significance was calculated by one-way analysis of variance (ANOVA). *p＜0.05, **p＜0.01, ***p＜0.001.


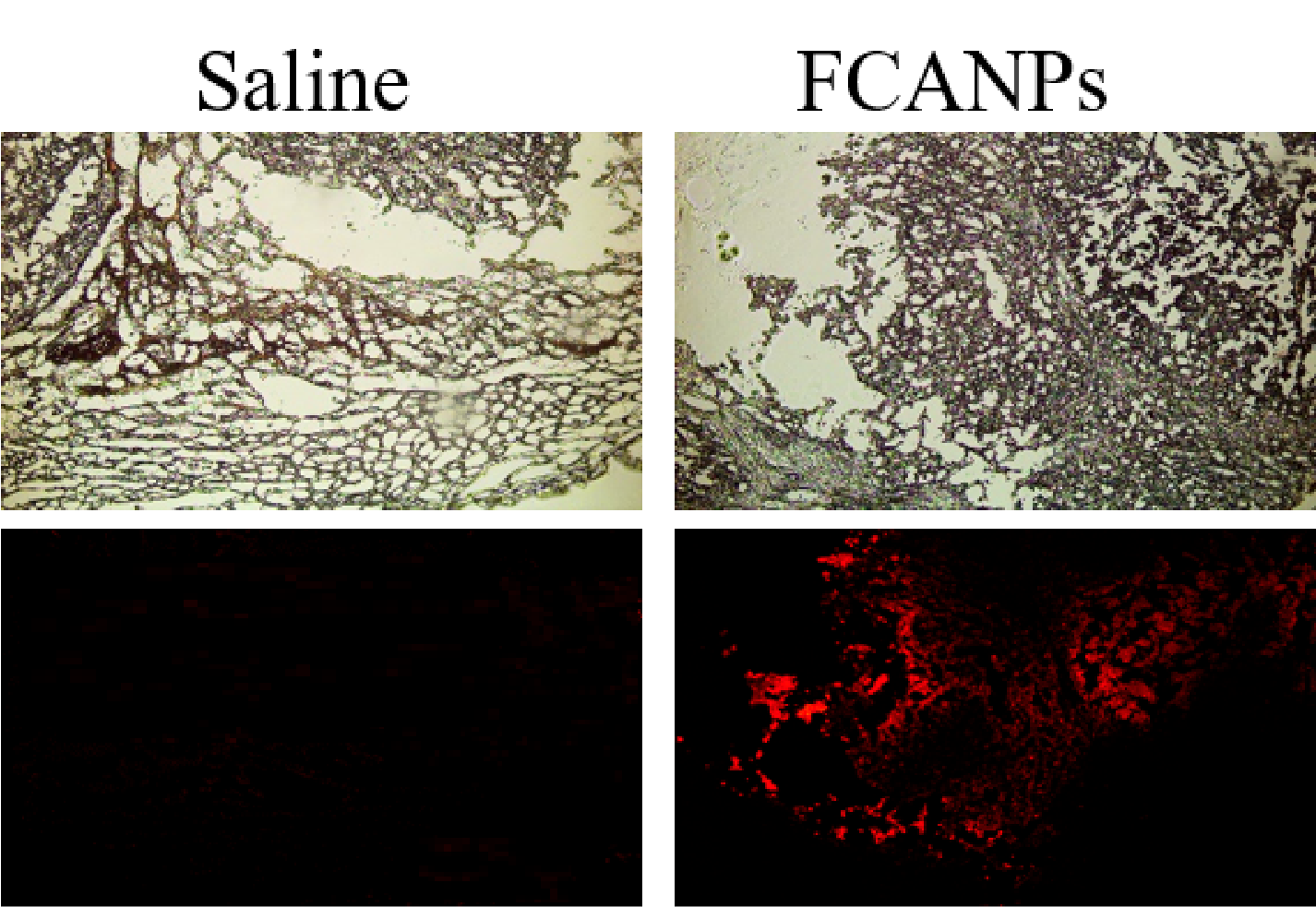


Figure S6. Histological and fluorescent images of frozen tumor sections (10 µm thick). Cy-5.5 fluorescence signal was shown in red.


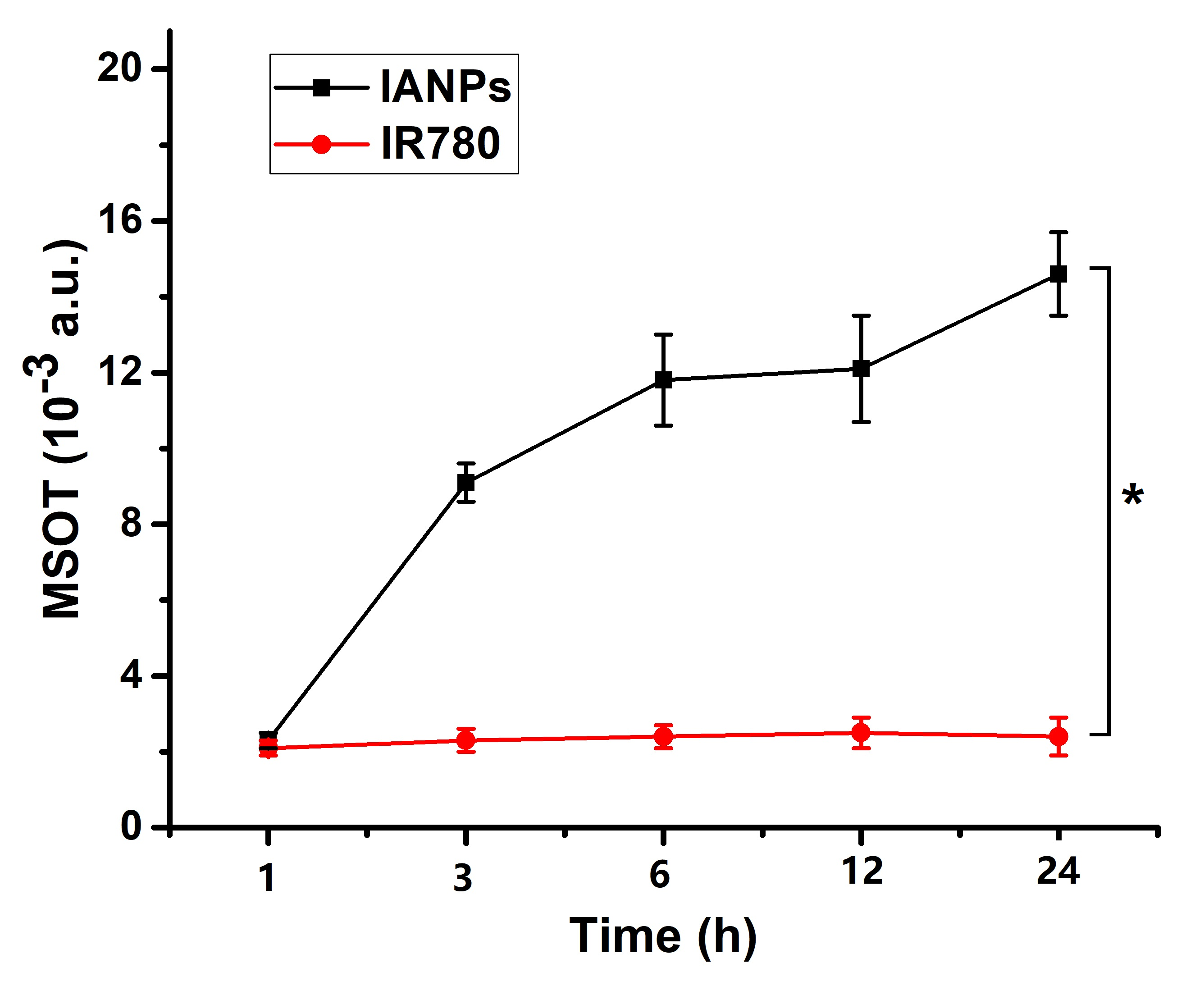


Figure S7. Quantitative analysis of relative PA intensity of IR780 in xenograft tumors at indicated time points after intravenous administration of IANPs and free IR780. data are shown as mean ± SD, n = 3. Statistical significance was calculated by performed by paired Student’s t test. *p＜0.05,


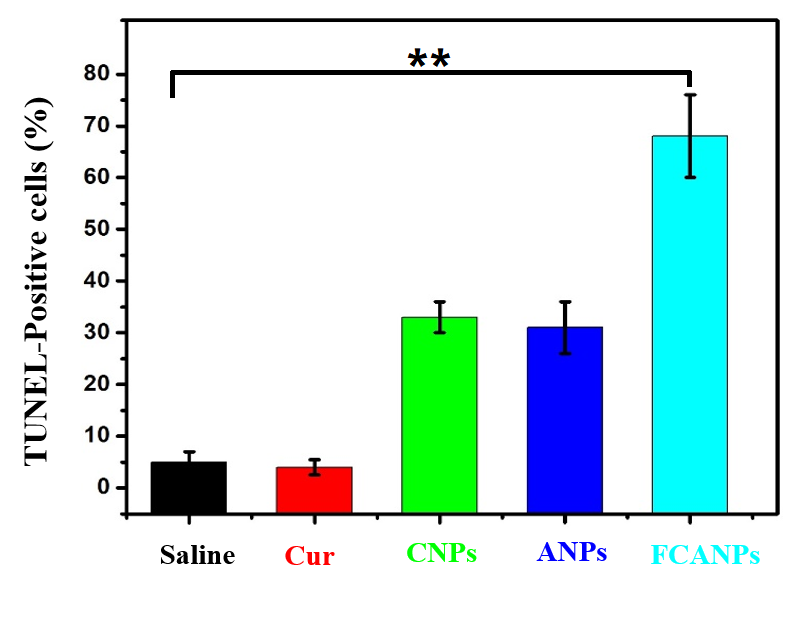


Figure S8. TUNEL-positive cells (%) in tumors of each group. data are shown as mean ± SD, n = 6. Statistical significance was calculated by one-way analysis of variance (ANOVA). **p＜0.01,


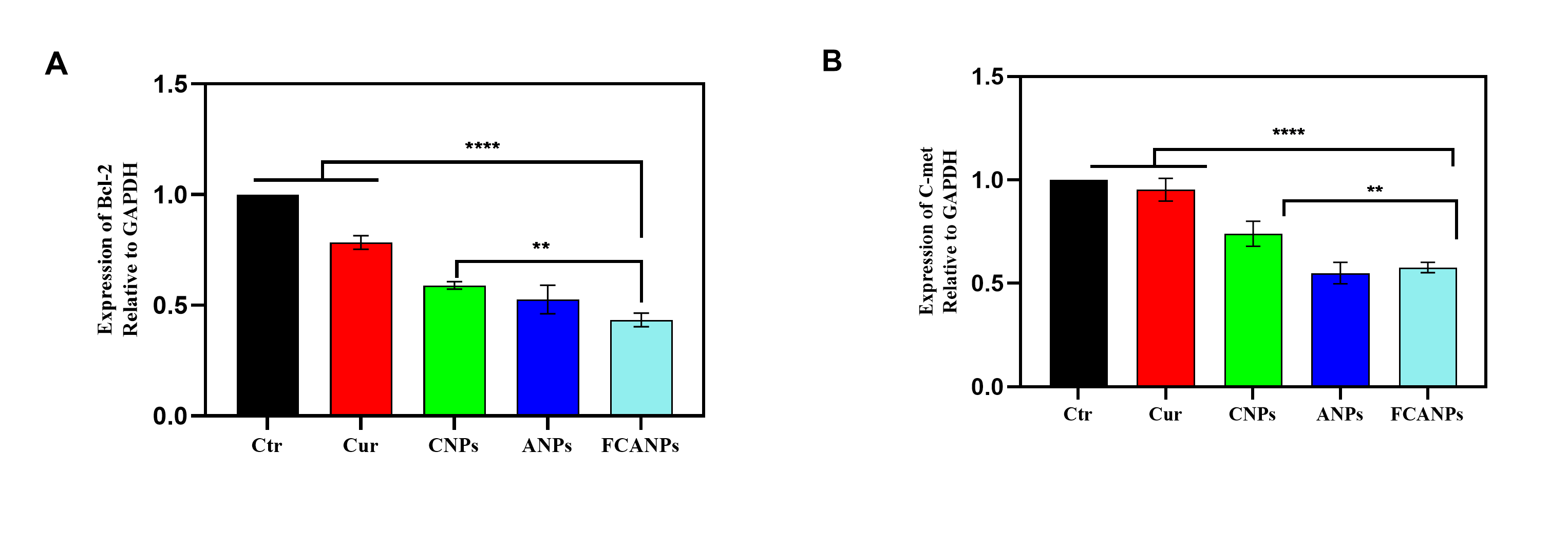


Figure S9. Relative C-met and Bcl-2 expression after different treatments by western-blot analysis. Relative band intensity was normalized by GAPDH. Data are shown as mean ± SD, n = 3. Statistical significance was calculated by one-way analysis of variance (ANOVA). **p＜0.01, ****p＜0.0001.
